# Genetic Engineering for Enhancing Sugarcane Tolerance to Biotic and Abiotic Stresses

**DOI:** 10.3390/plants13131739

**Published:** 2024-06-24

**Authors:** Tanweer Kumar, Jun-Gang Wang, Chao-Hua Xu, Xin Lu, Jun Mao, Xiu-Qin Lin, Chun-Yan Kong, Chun-Jia Li, Xu-Juan Li, Chun-Yan Tian, Mahmoud H. M. Ebid, Xin-Long Liu, Hong-Bo Liu

**Affiliations:** 1 National Key Laboratory for Tropical Crop Breeding, Sugarcane Research Institute, Yunnan Academy of Agricultural Sciences, Yunnan Key Laboratory of Sugarcane Genetic Improvement, Kaiyuan 661699, China; tanweerkm_biologist@outlook.com (T.K.);; 2 Sugar Crops Research Institute, Agriculture, Fisheries and Co-Operative Department, Charsadda Road, Mardan 23210, Khyber Pakhtunkhwa, Pakistan; 3 National Key Laboratory for Tropical Crop Breeding, Institute of Tropical Bioscience and Biotechnology, Chinese Academy of Tropical Agricultural Sciences, Sanya 572024, China; 4 Sugar Crops Research Institute, Agricultural Research Center, Giza 12619, Egypt

**Keywords:** sugarcane, biotic and abiotic stress, genetic engineering, transgenic sugarcane, genome editing

## Abstract

Sugarcane, a vital cash crop, contributes significantly to the world’s sugar supply and raw materials for biofuel production, playing a significant role in the global sugar industry. However, sustainable productivity is severely hampered by biotic and abiotic stressors. Genetic engineering has been used to transfer useful genes into sugarcane plants to improve desirable traits and has emerged as a basic and applied research method to maintain growth and productivity under different adverse environmental conditions. However, the use of transgenic approaches remains contentious and requires rigorous experimental methods to address biosafety challenges. Clustered regularly interspaced short palindromic repeat (CRISPR) mediated genome editing technology is growing rapidly and may revolutionize sugarcane production. This review aims to explore innovative genetic engineering techniques and their successful application in developing sugarcane cultivars with enhanced resistance to biotic and abiotic stresses to produce superior sugarcane cultivars.

## 1. Introduction

Sugarcane (*Saccharum* spp. hybrids) is a major tropical and sub-tropical crop cultivated in over 121 countries across 27 million hectares of land worldwide. It contributes about 80% of the world’s total sugar production and is the most efficient feedstock for bio-ethanol and diesel, accounting for 40% of the world’s biofuel production [1]. In addition, sugarcane is the source of valuable products such as paper, acetic acid, plywood, fibers, bio-fertilizer, animal feed, board, and industrial enzymes [2]. Conventional breeding methods for sugarcane aim to develop new hybrid varieties with higher yields and increased sugar contents [3]. However, the high genetic complexity of sugarcane hybrids, and a variable number of chromosomes (ranging from 53 to 143 chromosomes with an estimated genome size of 10 Gb pose significant challenges to the breeders [4]. At the same time, conventional breeding is costly, labor-intensive, and time-consuming, taking up to 10–15 years to release a new elite variety [5]. To acquire more of the intricate nature of the sugarcane genome, numerous initiatives have led to a variety of genome sequencing projects, encompassing parental species and hybrid genotypes [6,7,8,9,10,11,12,13]. More recently, the Chinese Academy of Sciences has initiated a pilot program in collaboration with the National Key Laboratory project, specifically targeting tropical crop breeding. Such scientific collaboration is anticipated to yield significant outcomes and contribute positively to the field of sugarcane genetic improvement in the future. 

**Figure 1:**
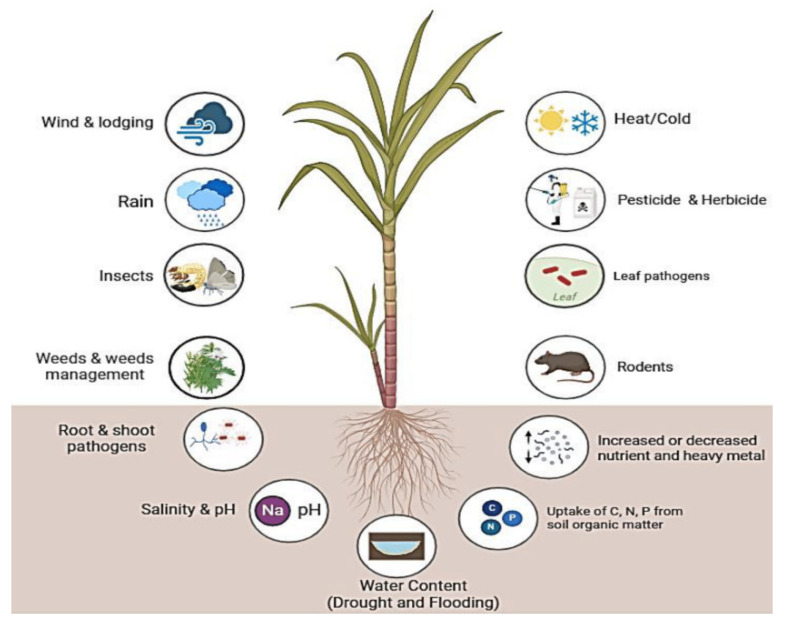
Various abiotic and biotic stress affecting sugarcane plant growth, development, and productivity. (https://biorender.com; accessed on 23 April 2024).

On the other hand, sugarcane cultivation is adversely affected by biotic and abiotic stresses, including weeds, diseases, insects and pests, drought, salinity, low temperatures, heavy metals, and low soil fertility (Figure 1). More importantly, genetic engineering and genome editing have emerged as powerful tools to address these challenges and increase yield productivity without losses caused by these stresses. Sugarcane is a vegetatively propagative crop and breeding aspects make it an excellent candidate for crop improvement through genetic engineering, to produce green energy. Transgenic technology allows the precise transfer of one or more stacks of genes from unrelated plants or different organisms. Since the 1990s, different genetic transformation techniques have been successfully developed in sugarcane, including electroporation, *Agrobacterium tumefaciens*, the biolistic bombardment method, and so on [14,15]. In the following three decades, *Agrobacterium*-mediated transformation and other new methods have become routine exercises in many research laboratories worldwide. Moreover, genome editing can edit, insert, or replace specific sequences within the genome. A number of genes promoting resistance to biotic and abiotic stresses have been introduced into sugarcane for genetic improvement. Therefore, this review summarizes the advances in genetic improvement of sugarcane and its potential to increase yield productivity, especially under biotic and abiotic stresses.

## 2. Biotic Stress

### 2.1. The Enhancement of Herbicide Tolerance

**Figure 2:**
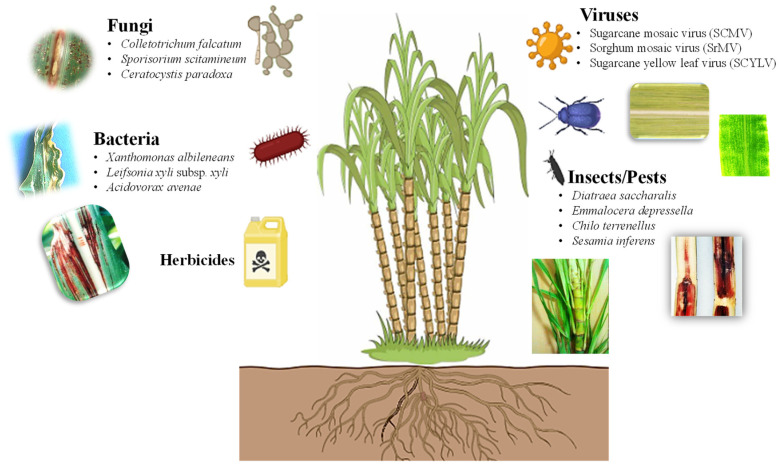
Types of biotic stresses that affect growth, yield, and productivity of sugarcane (https://biorender.com; accessed on 23 April 2024).

Due to a sessile nature, plants must confront various living organisms in the environment (Figure 2). Weed infestation is a serious problem that negatively impacts the yield of sugarcane, resulting in significant economic losses [16,17]. Introducing genes that confer herbicide tolerance into sugarcane can aid in the breeding of transgenic sugarcane tolerant to herbicides, reducing labor intensity and costs while ensuring a higher yield of sugarcane. Traditional breeding for herbicide-tolerant traits is lagging due to the lack of herbicide-tolerant gene pools in wild-sugarcane relative species [18]. An alternative strategy should be adopted for developing crops that are tolerant to broad-spectrum herbicides [19]. The advances in transgenic sugarcane that have been engineered for herbicide tolerance are summarized in Table 1.

The integration of the *bar* gene into the genome of sugarcane using the biolistic transformation method resulted in the production of herbicide-tolerant transgenic sugarcane [20]. Using *Agrobacterium*-mediated transformation to introduce the herbicide-tolerant genes *bar* and *CP4-EPSPS* into the main sugarcane varieties, resulted in transgenic sugarcane exhibiting excellent herbicide tolerance [19,31]. By incorporating the genome of embryonic calli with a glyphosate-tolerant (*GT*) gene, the first study published the genetic transformation of four sugarcane genotypes through the bombardment of embryonic calli with the *GT* gene. These results showed that 88% of the transgenic plants survived on the first application of glyphosate, whereas all the non-transformed plants died. In the second application, increasing the dose of glyphosate resulted in the accumulation of *GT* gene-encoded products that survived [22]. However, transgenic glyphosate-tolerant sugarcane was developed for commercial release through the genetic transformation of cultivar RA87-3. The objective was to obtain glyphosate-tolerant transgenic events in two recently released cultivars, TUC 95-10 and TUC 03-12, by introducing *EPSPS* and *nptII* genes using the microprojectile method. A transgenic event tolerant to the glyphosate herbicide was successfully obtained through the biolistic transformation using the TUC 03-12 genotype as the starting material. The resulting genotype showed greater than 99% similarity with the parental genotype [23,32].

However, sugarcane borers and weeds threaten production, quality, and yield. Genetic modification using stack genes technology (combining two or more genes) was performed by introducing modified cane borer-tolerant (*CEMB-Cry1Ac*) and glyphosate-tolerant (*CEMB-GT*) genes into sugarcane. Transgenic plants showed efficient tolerance to cane borers and tolerance to glyphosate, indicating sustainable tolerance in sugarcane [24]. A transgenic sugarcane cultivar (ROC22) was regenerated using herbicide screening, containing the *bar* gene to obtain sense and antisense strigolactones biosynthesis genes (*ScD27.2*). All transgenic lines tested positive for herbicide tolerance and contained the target gene. Furthermore, PCR detection and 1% Basta (Glufosinate) application on leaves revealed *bar* in all lines [25]. A recent study has presented an efficient transformation system mediated by *Agrobacterium*, which utilizes the herbicide-tolerant *bar* gene as the selectable marker. The transgenic lines were confirmed through PCR and exhibited tolerance to herbicides. Moreover, the protocol demonstrated several advantages, including a higher yield of transgenic lines from calli, improved selection, and increased transformation efficiency [26]; these results showed that a dependable tool now offers a standard procedure for cultivating robust transgenic sugarcane plants. 

Additionally, gene editing technology has provided new strategies for breeding herbicide-tolerant sugarcane varieties. CRISPR/Cas9-mediated multi-allelic gene mutation techniques mutate the acetolactate synthase gene (*ALS*) to develop broad-spectrum herbicide-tolerant sugarcane plants [33]. The development of environmentally friendly herbicide-tolerant sugarcane varieties through genetic engineering and gene editing technology will help to reduce the use of harmful herbicides and make sugarcane cultivation more environmentally friendly and sustainable in the future.

### 2.2. Enhancing Disease Tolerance in Sugarcane

The defense mechanisms of sugarcane are triggered in response to biotic stressors, including different pathogens, such as fungi, bacteria, viruses, and insects [34,35,36,37,38]. These biotic stressors result in serious diseases in sugarcane (Figure 2), such as red rot (*Colletotrichum falcatum*), smut (*Sporisorium scitamineum*), pineapple disease (*Ceratocystis paradoxa*), red stripe (*Acidovorax avenae*), leaf scald (*Xanthomonas albileneans*), grassy shoot disease (*Phytoplasma*), and mosaic virus (ScMV) and yellow leaf virus (ScYLV) infection, as well as sugarcane stem borer (*Diatraea saccharalis*), African sugarcane stalk borer (*Eldana saccharina*), sugarcane weevil (*Sphenophorus levis*), and various pests such as borers, pyrilla, thrips, grasshoppers, sucking pests, and cane grubs (*Melanaphis sacchari*) [16,39,40]. Sugarcane crops are susceptible to over 100 different types of pathogens [41]. Breeding programs play a crucial role in genotype screening for disease resistance. However, sugarcane breeders face significant challenges simultaneously introducing resistance to all pathogens [42]. Therefore, biotechnological strategies have been developed to produce commercial clones with superior agronomic traits; these genetic modifications are summarized in Table 2.

The most prevalent viral diseases affecting sugarcane crops include sugarcane mosaic virus (SCMV), Sorghum mosaic virus (SrMV), and yellow leaf syndrome caused by Sugarcane yellow leaf virus (SCYLV) [41,57,58]. To address this, the preliminary virus capsid protein (CP) was utilized to produce transgenic plants that are immune to viruses. These plants produce a viral protein that co-suppresses and bypasses several stages of the viral life cycle and reduces disease manifestation. The use of untranslated SrMV strain *CP* enabled the cultivation of transgenic sugarcane plants. These plants display complete susceptibility to fully tolerant phenotypes [43]. In another study, transgenic lines with enhanced tolerance to Fiji disease virus (FDV) were obtained using particle bombardment. The transgene encodes a translatable version of FDV segment 9 of 1 open reading frame (ORF) of the virus genome. The tolerance of the transgenic plants was evaluated using a glasshouse experiment. However, the phenotypes of transgenic plants were not entirely consistent with resistance mechanisms based on post-transcriptional gene silencing (PTGS) [44].

Transgenic sugarcane against SCYLV was developed through the biolistic bombardment of cell cultures with an untranslatable *CP* gene. The resistance level exhibited by some transgenic plants was comparable to that of the completely resistant cultivar. These results were based on the virus titer and disease symptom development [45]. A strong innate defensive response against viral infections has been demonstrated using RNA interference (RNAi). In the host defense system, viruses produce suppressors of the host RNA interference (RNAi) pathway. A potential approach to studying viral pathogenesis and managing mosaic disease in sugarcane may involve identifying the microRNAs (miRNAs) encoded by the sugarcane streak mosaic virus (SCSMV) and further requires understanding the host genes that are related to miRNA targets [59]. Moreover, the study examined the resistance of transformed plants to the SCMV, using both the full 927 base pairs (bp) of the *CP-SCMV* gene sequence and truncated sequences of 702 bp. These results demonstrated that plants with the complete gene sequence exhibited a more protective strategy against the virus than those with truncated sequences [46]. Furthermore, the expression of short hairpin RNAs (shRNAs) confers resistance against SCMV infection [1,47]. These findings validated that RNAi technology can confer resistance against SCMV and the ubiquitin promoter is effective in the development of transgenic sugarcane lines [60].

Moreover, a study was conducted to evaluate the agronomic performance and viral resistance of transgenic lines transformed with SCYLV resistance, using antisense expression of a portion of the viral *CP* gene. The parental genotype outperformed the transgenic lines in sugarcane yield. However, the transgenic lines displayed lower SCYLV infection (0–5%) than the parental variety (98%). These variations in yield could be attributed to somaclonal variation during in vitro regeneration of transgenic plants. These findings suggest that transgenic sugarcane exhibits variable agronomic performance and disease resistance [48,49]. These results warrant further investigation to develop more efficient and reliable transgenic sugarcane cultivars. The use of transgenic sugarcane lines expressing the *SCMV-CP* gene led to a higher yield than the wild-type, with all evaluated lines producing significantly more cane and sucrose per hectare than the parental variety. In addition, transgenic lines exhibited a lower incidence of SCMV than the parental variety. These findings indicated that the introduction of transgenic sugarcane expressing *SCMV-CP* could be a viable solution for enhancing agronomic traits and viral disease resistance [61]. Taken together, these studies demonstrated that transgenic plants undergo minor hitherto discernible changes in morphology, physiology, and phytopathology despite low levels of genomic changes. Therefore, it is essential to assess somaclonal variation within transgenic populations to ensure the proper management and evaluation of transgenic sugarcane for field trials [62,63]. 

A comparative study aimed to evaluate the efficacy of two transgenic sugarcanes, generated through Pathogen-derived resistance (PDR) and RNAi methods using gene-encoding coat protein (CP) of SCMV, against SCMV through artificial viral inoculation. These results indicate that the RNAi approach targeting the CP gene proves more effective in producing resistance against SCMV infection in transgenic sugarcane compared to the PDR approach [50]. These results highlight promising strategies for developing SCMV-resistant sugarcane through genetic engineering. Breeding virus-resistant sugarcane varieties is the main goal of the sugarcane breeding program. Double-stranded RNA-specific ribonuclease (*PAC1*) encoded by the *Pac1* gene from *Schizosaccharomyces pombe* was introduced into a virus-sensitive sugarcane cultivar by *Agrobacterium*-mediated transformation. These results showed that transgenic plants possess significantly milder symptoms and lower viral loads than the wild-type, providing a basic foundation for breeding virus-resistant sugarcane [51].

To combat fungal diseases such as brown rust in sugarcane, glucanase and chitinase genes have been expressed in transgenic sugarcane plants. Transgenic sugarcane resistant to red rot was developed by overexpressing the *β*-*1*,*3*-*glucanase* gene from *Trichoderma* spp. as the primary target of fungal attack. Microscopic examination of parenchyma cells in the stalks of these plants was filled with sucrose, which inhibited the growth of *C. falcatum* hyphae. Transgene overexpression after infection was upregulated and successfully transmitted to the second generation of clones [52]. Moreover, the potential of transgenic sugarcane lines expressing the barley *chitinase class II* gene against *C. falcatum* infection was assessed. Protein extracts from transgenic sugarcane plants inhibited the growth of *C. falcatum* mycelia, and transgenic lines demonstrated strong resistance against *C*. *falcatum*. Additionally, the mRNA expression of the transgene in *C. falcatum* inoculated transgenic sugarcane lines was outperformed by control parental plants [53]. Two genes, *SUGARWIN1 (SWO*) and *SUGARWIN2 (SWT*), were overexpressed in sugarcane to improve resistance against *C. falcatum*. Transgenic plants showed significant inhibition of the fungal pathogen infection and proliferation, indicating potential for developing crops with better fungal resistance [54]. Smut disease, caused by *S. scitamineum*, is considered a destructive disease of sugarcane. Rice receptor-like cytoplasmic kinase, known as broad-spectrum resistance 1 (*BSR1*) in sugarcane, confers resistance against smut under greenhouse conditions. *BSR1* overexpressing line displayed normal growth and morphology [55], suggesting that *BSR1* overexpression is effective in conferring broad-spectrum disease resistance in the different crops.

### 2.3. The Reinforcement of Insect and Pest Resistance

Insects and pests cause significant reduction in the yield and quality of sugarcane using different stem borers (Figure 2), such as Lepidoptera (*D. saccharalis*), root borers (*Emmalocera depressella*), top borers (*Chilo terrenellus*), pink borers (*Sesamia inferens*), and pink stem borers (*Sesamia cretica*) [64,65]. The extensive use of pesticides can harm other life on land such as beneficial insects, vertebrates, humans, and the environment. Pesticides may also pollute different soil zones before reaching deep water levels. Modern biotechnological approaches have made rapid progress in the genetic engineering of sugarcane plants to protect against pests by transferring genes derived from plants, pests, and bacteria [66,67,68] (Table 3).

Alternatively, the genetic transformation of several insecticidal proteins, such as lectins and protease inhibitors (PIs), has been used in genetic transformation. Other genes included *avac* (*Amaranthus viridis* L. agglutinin), *skti* (soybean Kunitz trypsin inhibitor), *sbbi* (Bowman–Birk inhibitor), and *gna* (*Galanthus nivalis* agglutinin) [69,70]. Transgenic sugarcane plants expressing either potato proteinase inhibitor II or the snowdrop *lectin* gene showed increased antibiosis in the larvae of the canegrub *Antitrogus consanguineus* under glasshouse conditions. Canegrubs feeding on the transgenic line transformed with the potato gene showed 4.2% weight gain in canegrubs fed on control plants. Similarly, larvae feeding on the roots of transgenic lines transformed with the snowdrop gene showed a 20.6% weight gain of grubs feeding on wild-type plants. In contrast, larvae from the Greyback Cane Beetle (*Dermolepida albohirtum*) fed on transgenic plants expressing snowdrop lectin and proteinase inhibitor decreased in weight compared to larvae fed on non-transformed plants [18]. However, the growth of *D. saccharalis* larvae fed on transgenic sugarcane transformed with *skti* and *sbbi* from soybean was restricted compared to that of larvae from control plants [20]. In vivo bioassay studies of transgenic sugarcane transformed with a synthetic bovine pancreatic trypsin inhibitor (*aprotinin*) gene against the top borer (*Scirpophaga excerptalis*) indicated that larvae fed on transgenic plants exhibited a decrease in larval weight of up to 99.8% [71]. Transgenic sugarcane plants overexpressing sugarcane cysteine peptidase inhibitor 1 (*CaneCPI-1*) were evaluated for their potential resistance through feeding assays with larvae of sugarcane billbug (*S. levis*); significantly less damage by larval attack has been observed in transgenic plants than non-transgenic plants [66]. The trypsin inhibitory activity of PIs from *Erianthus arundinaceus*, a wild-sugarcane relative, was evaluated and showed significant differences among different plant tissues. The highest inhibitory activity was found in meristematic tissue, and PIs isolated from the apical meristem effectively inhibited the mid-gut proteinases of early shoot borer (*C. infuscatellus*) and internode borer (*Chilo sacchariphagus indicus*) [91,92]. These studies have demonstrated the possibility of identifying new and efficient PIs for producing sugarcane plants that are resistant to stem borers. Furthermore, transgenic sugarcane lines displaying the Vip3A protein exhibited resistance to the sugarcane stem borer (*C. infuscatellus*). These results imply that incorporating a single copy of the *Vip3A* gene into transgenic sugarcane lines confers resistance to borers [72]. Consequently, these lines could potentially be used to produce insect-resistant transgenic sugarcane and could also be combined with the *Bacillus thuringiensis* (*Bt)* toxin in gene pyramiding to enhance resistance.

Nevertheless, the most important insecticidal proteins produced by the gram-positive spore-forming bacterium *B. thuringiensis* (*Bt*), which produces proteins during the vegetative phase (*Vip*) and sporulation phase (*Cry*), are toxic to a wide range of insects [73,93]. The development of transgenic *Bt* plants that are resistant to stem borers in sugarcane cultivation has been achieved using specific toxins. Studies have identified various *Bt* genes, including *cry1Ab*, *cry1Aa3*, *cry1Ac*, *s-cry1Ac*, *m-cry1Ac*, *cry2A*, and *vip3A*, that have been deployed (Table 3). More importantly, Bt proteins have been used to control sugarcane borer in maize for the past decade. However, practical use in sugarcane requires the delivery of high doses of protein and harbors the slow evolution of insect resistance. Two *Bacillus* proteins, *Cry1Ab* and *Cry2Ab*, have been expressed in commercial sugarcane varieties, demonstrating efficacy against sugarcane borer in the field. A strategy for trait deployment of tropical crops has also been described [74]. Moreover, the study aimed to develop transgenic sugarcane lines with increased resistance to the sugarcane borer *D. saccharalis.* The *Bt* genes were incorporated into embryogenic sugarcane calli. The transgenic lines with higher levels of *Bt* transcripts were identified and tested for resistance against the pest [18].

To explore the utilization of the *Pinellia pedatisecta agglutinin PPA* in controlling aphids in sugarcane, the resistance of independent transgenic sugarcane lines was assessed. Alterations in stomatal patterns, antioxidant enzymes, chlorophyll content, sugar, and tannins were examined before and after insect infestation. These findings revealed that *PPA* enhanced sugarcane resistance to sugarcane woolly aphids and insect resistance [75]. Taken together, there is an urgent need to fully utilize the recently sequenced sugarcane genome to investigate the interactions between sugarcane and its pathogens, as well as pathogens that are closely related to those affecting sorghum and other grasses sharing similar genomes [6,7,8,9,10,12,13]. However, it is expected that the reference genome for *Saccharum* spp. hybrids is still underway because of its complexity and high polyploidy, which may serve as a tool for searching resistance genes for many common diseases in sugarcane, and functional disease resistance will be maintained when genes are moved across the species using transgenic technology.

## 3. The Enhancement of Tolerance to Abiotic Stress

**Figure 3:**
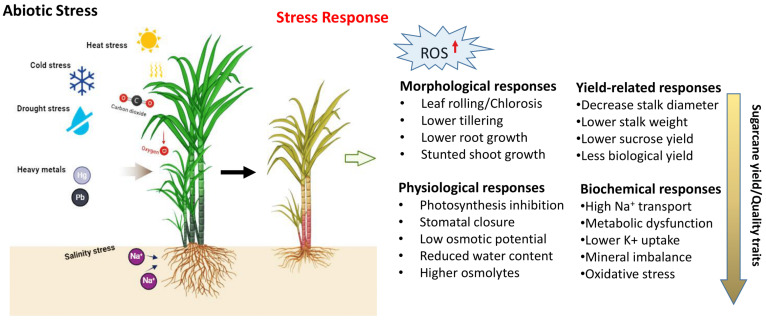
Types of abiotic stresses and stress responses that affect yield productivity of sugarcane (https://biorender.com; accessed on 23 April 2024).

Sugarcane cultivation is influenced by a variety of abiotic factors, such as drought, salinity, high temperature, nutrient deficiencies, and heavy metals, which significantly decrease the average yield (Figure 1). To cope with adverse environments, plants must adopt tolerance mechanisms and undergo various morphological, anatomical, physiological, and cellular changes in response to abiotic stresses [94]. The plant response to stress is a multifaceted process that involves various signaling pathways, post-translational modifications, secondary metabolite synthesis, nitrogen metabolism, and the activity of various proteins, including transcription factors, kinases, and transporters. The genes responsible for regulating biotic and abiotic stress responses may function cumulatively or redundantly, activating both common and distinct downstream targets [95]. Adapting to these stresses is also linked to metabolic adaptation, which results in the accumulation of various organic solutes like proline, sugars, betaines, and polyols. To protect from the harmful effects of reactive oxygen species (ROS) (Figure 3), plants utilize antioxidant enzymes such as ascorbate peroxidase APX, SOD, and GR, and non-enzymatic antioxidants like carotenoids, ascorbic acid, and flavonoids [96,97]. Researchers have identified different genes that contribute to abiotic stress tolerance (Table 4) in model/water-tolerant plants, unrelated plants, or completely different organisms, including those involved in the overexpression of transcription factors (TFs) and effector proteins, which play crucial roles in activating genes in response to abiotic stresses [98,99].

The COR/DREB (Cold-induced regulated-dehydration-responsive binding element) family of regulatory proteins is the first known set to play a role in the regulation of abiotic stress genes in plants [113]. Studies have shown that the overexpression of *Arabidopsis AtDREB2A* in transgenic sugarcane can enhance drought tolerance under greenhouse conditions. In these experiments, transgenic plants exposed to drought stress exhibited higher relative water content (RWC), carbon assimilation, sugar content, and bud sprouting, without affecting biomass [100]. B-box (BBX) proteins are essential for the control of plant growth and development. Transgenic sugarcane overexpressing the *AtBBX29* gene exhibited improved rates of photosynthesis and higher levels of antioxidants and osmolytes under drought conditions [101]. In another study, the accumulation of osmolytes, such as proline, soluble sugars, and glycine betaine, was observed in sugarcane plants engineered to overexpress the tomato ethylene-responsive factor gene (*TERF*), and these transgenic plants displayed reduced ROS and malondialdehyde (MDA) content [2].

The *Arabidopsis* H^+^-pyrophosphatase type I gene (*AVP1*) is involved in the control of apoplastic pH and auxin transport, and overexpression of the *AVP1* gene in sugarcane results in increased RWC and osmotic and turgor potential in the leaves, indicating that improved drought and salinity tolerance. In addition, transgenic sugarcane plants exhibited profuse rooting systems [102,103]. Proapoptotic BAX proteins are responsible for controlling programmed cell death (PCD). Overexpression of the BAX inhibitor gene (*BI-1*) from *A. thaliana* in sugarcane plants increases drought tolerance by reducing the induction of cell-death pathways [104]. The drought-responsive gene *BRK1* from *S. spontaneum* was transformed into sugarcane, and *BRK1* transgenic lines had improved physiological parameters. Interlocking marginal lobes in epidermal leaf cells were observed in all transgenic *BRK1* lines during drought stress compared with the wild-type. These findings suggest that *BRK1* plays a potential role in sugarcane’s response to drought stress, promoting leaf epidermal cell morphogenesis and actin polymerization [114].

Plants exposed to salinity stress acquire osmoprotectant molecules, such as proline, which serve not only as a source of nourishment but also as scavengers such as ROS, to maintain cellular activities. By overexpressing the pyrroline-5-carboxylase synthase gene *(P5C5*), salinity-tolerant sugarcane was obtained [105]. Additionally, improved tolerance to water deprivation was achieved through overexpression of a related gene (*SoP5C5*). *P5C5* plays a crucial role in proline production. The ROS-scavenging glyoxalase pathway enzymes, including glyoxalase I (Gly I), glyoxalase II (Gly II), and glyoxalase III (Gly III), help transgenic sugarcane plants to eliminate harmful compounds, such as glyoxylate, under abiotic stress conditions [106]. Furthermore, under salt stress, transgenic sugarcane overexpressing the *EaGlyIII* gene displayed improved RWC, photosynthesis, osmolytes, and ROS-scavenging enzyme activities, resulting in increased biomass and enhancing germination rates [107]. These results indicate that the overexpression of *EaGly III* could be a promising approach for salt and drought tolerance in sugarcane [115]. However, the *ScD27.2* gene was introduced into sugarcane to study its role in drought tolerance. The results showed that interfering with *ScD27.2* expression decreased tolerance to drought stress, indicating its role in sugarcane growth and development [25].

Plant cells respond to stressful conditions by producing a family of stress proteins known as heat shock proteins (HSPs). In *E. arundinaceus*, a sweet cane, *HSP70* overexpression has a strong protective effect when plants are exposed to water and salt stress. Transgenic plants displayed increased photosynthetic efficiency, RWC, stress-induced gene expression, and cell membrane thermostability [108]. Transgenic sugarcane with the drought-tolerant *Ea-DREB2B* gene from *S. arundinaceum*, a wild species of *S. officinarum,* can regulate response to drought stress. In addition, interactions between soil fungi and bacterial communities in the rhizoplane, rhizosphere, and bulk soil were assessed. The results revealed that the host plant genotype plays a crucial role in strengthening plant–fungi interactions and enhancing beneficial fungal function in the root-related area of transgenic sugarcane, allowing response to drought stress. Moreover, changes in the soil environment caused by transgenic sugarcane alter bacterial communities [116,117].

However, limited studies have been conducted on cold-tolerant transgenic sugarcane (Table 4). These plants overexpress the bacterial isopentenyl-transferase gene (*ipt*) using the cold-inducible promoter *AtCOR15* derived from *A. thaliana*. Compared with non-transgenic parent plants exposed to low temperatures, transgenic sugarcane exhibited increased chlorophyll, reduced MDA content, and decreased electrolyte leakage [109]. Transgenic plants overexpressing the tubulin gene (*TUA*), cloned from cold-tolerant sugarcane varieties, enhanced the cold tolerance of cold-susceptible sugarcane varieties, exhibiting increased soluble protein and sugar content, enhanced peroxidase activity, and reduced MDA content compared to non-transgenic plants [110]. Further investigation into the effects of cold stress could have substantial implications for the sugarcane industry, as cold stress negatively affects sugarcane crop yields and quality.

## 4. Production of New Compounds and Renewable Energy Sources

Genetically modified sugarcane has achieved significant improvement under biotic and abiotic stress conditions. Plant cells, as opposed to animal cells, are cost-effective and safe methods for producing new recombinant (r) proteins. Sugarcane plants have well-developed storage tissue systems, which make them promising candidates to produce high-value compounds and pharmaceuticals. Recent advancements in bio-pharming have enabled the production of a wide range of essential products, such as high biomass production, rapid growth, and efficient carbon fixation, making sugarcane more attractive for novel compound applications [41,57,118]. The utilization of sugarcane for molecular farming has gained significant momentum owing to its minimal transgene dispersal and high amount of extractable juice with low protein content. The culm of sugarcane comprises 70% dry weight and is predominantly composed of parenchyma cells, a desirable target for r-protein production. Within plant cells, vacuoles constitute a large portion of the cell volume, contributing 60% of the cane weight [57,119]. Thus, targeting r-proteins in the vacuole can result in high-protein yields and relatively simple purification.

Initial attempts were made to express proteins in the cytoplasm and purify from the leaves, resulting in low yields due to the presence of numerous other proteins that may interfere with downstream processing [120,121]. However, targeting proteins to vacuoles in the stem parenchyma may lead to higher levels of recombinant proteins. Previous studies have shown that newly identified vacuolar-targeting determinants (VT) of 78-bp or 18-bp in combination with strong constitutive promoter (Port ubi882) have proven to be effective in the production of large quantities of r-proteins. However, the presence of vacuolar proteolytic enzymes and the acidic nature of lytic vacuoles must be considered when selecting recombinant candidate proteins. It is estimated that 4.8–7.2 kg of r-protein can be produced per acre (40,000 canes per acre) with a purity of 50% after affinity chromatography [122]. This yield is comparable to commercially available r-proteins. The abundance of extractable juice with low protein content and well-established juice processing technology has motivated researchers to focus on the culm of sugarcane. Thus, culms have drawn attention due to their significant biomass output and potential for the large-scale production of recombinant proteins. Sugarcane could be an effective platform to produce recombinant proteins, and further research could significantly impact the biotechnology sector. Taken together, targeting r-proteins to the lytic vacuoles of sugarcane shows promising results in the production of recombinant proteins. Sugarcane can extract a large volume of juice coupled with newly identified vacuolar-targeting determinants and strong constitutive promoter, making it an efficient platform for molecular farming.

Trehalose is a naturally occurring non-reducing disaccharide that plays an important role in regulating carbon metabolism and photosynthesis. To investigate the effects of trehalose synthesis on sucrose accumulation in sugarcane, overexpression of *Escherichia. coli, otsA* (trehalose-6-phosphate synthase TPS), *otsB* (trehalose-6-phosphate phosphatase; TPP), and silenced native *TPS* expression altered the effect of trehalose synthesis. These findings suggest that manipulating Tre6P/trehalose metabolism could be a potential strategy for modifying the sugar profile of sugarcane stems [123]. Sucrose phosphate synthase (*SPS*) is a vital enzyme that plays a key role in regulating sucrose content in sugarcane by controlling the synthesis of sucrose. Enhancing *SPS* activity may be a useful approach to increasing sugarcane yield [124]. The effects of overexpressing the *SoSPS1* gene on sucrose accumulation and carbon partitioning in transgenic sugarcane were studied. Overexpression of *SoSPS1* resulted in increased levels of sucrose and enhanced enzymatic activity in the leaves and stalks, leading to improved plant growth [125]. Transgenic sugarcane with enhanced sucrose yield was developed by the over-expression (*SoSPS1*) gene. Furthermore, the nutritional and mineral composition of transgenic lines and non-transgenic sugarcane were analyzed after 11 months in field conditions. Protein and potassium content was higher in stems of transgenic lines. However, wild-type and transgenic sugarcane were found to be substantially equivalent in terms of nutritional and mineral compositions [126]. The development of new sucrose isomers, trehalulose and isomaltulose, is facilitated by the insertion of sucrose isomerase genes. The transgene expression in the sugarcane culms exhibited significantly greater expression levels than in leaf tissues. The overall sugar estimation conducted in internodes of the transgenic sugarcane revealed increased sugar concentrations in mature sugarcane culms. The transgenic sugarcane lines demonstrated the highest sugar recovery of 14.9%, compared to 8.5% in the control lines [127]. However, consistent attempts to modify sugar metabolism to increase sugar yield remain challenging for molecular farming.

Another field of study is bioplastics such as polyhydroxyalkanoates (PHAs), which are biodegradable. Previous studies have shown that these polymers can be produced from sugarcane, including polyhydroxybutyrate (PHB) [128,129]. Utilization of sugarcane as a renewable bioenergy crop is a new emerging task. The selection of superior multipurpose cultivars for agro-industries is based on genetic and biotechnological strategies. To meet the demand for renewable energy sources, sugarcane has received numerous genes promoting resistance to biotic and abiotic stresses [41,57,118]. Nonetheless, considerable emphasis has been placed on improving the production of bioenergy and biofuel. Various approaches have been devised to modify lignin, facilitate saccharification, and enhance second-generation bioethanol [130,131]. Recently developed transgenic sugarcane, termed oilcane, has been genetically engineered to direct carbon flux towards biosynthesis and accumulation of energy-rich triacylglyceride (TAG) molecules in vegetative tissues [132]. This oilcane was employed to produce five high-value bioproducts: hydroxymethylfurfural (HMF), furfural, acetic acid, fermentable sugars, and vegetative lipids [132,133,134]. Genetic and biotechnological strategies have been employed to improve sugarcane productivity, including the development of transgenic sugarcane that directs carbon flux toward the biosynthesis and accumulation of energy-rich molecules. These advances in sugarcane biotechnology hold great potential for the development of future energy sources for biofuel production. Conversely, the success of these developments depends on the careful selection of techniques employed for sugarcane transformation, choice of promoters and marker genes, target tissue/explants, and tissue culture system (Table 1, Table 2, Table 3 and Table 4). Further research and development in sugarcane biotechnology will be crucial for meeting the increasing demand for renewable energy sources and sustainable agro-industries. However, little progress has been made in cultivating sugarcane for commercialization through genetic engineering in a few countries (Table 5).

## 5. Status and Concerns about GM Sugarcane

Since its inception, the debate over genetically modified organisms (GMOs) has continued, with a particular focus on the release of herbicide-tolerant sugarcane and its potential impact on the sugar export market. Several studies have been conducted on sugarcane using various genetic transformation techniques. For example, the safety of transgenic sugarcane with the *AVP1* gene [103] was assessed through animal feeding and genotoxicity assays. Acute and subchronic toxicity studies were conducted in rats, and no significant differences were observed among the different treatments [135]. Moreover, genotoxicity assessment revealed that GM sugarcane was neither genotoxic nor cytotoxic to rats. These results suggest that GM sugarcane is non-toxic to experimental animals and is suitable for commercial release. A field study conducted on transgenic sugarcane expressing insect and glyphosate tolerance genes showed poor agronomic and industrial traits compared to non-transformed plants [21]. Some studies have reported successful field trials on disease-resistant transgenic sugarcane varieties. For instance, two cultivars resistant to SCMV showed large variations in yield and disease resistance in the field condition [48]. In another trial, transgenic sugarcane lines transformed for SCYLV resistance showed reduced performance compared to the parental genotype, despite their viral resistance [49]. Conversely, a positive result was observed in a field experiment conducted on transgenic sugarcane lines expressing the *CP* gene of SCMV, with greater yield and lower SCMV disease incidence at four different experiment locations in China across two successive growing seasons [61]. However, only a few officially approved varieties (ISAAA 2024: Table 5) have been released.

A transgenic trait expressing the herbicide-resistant sugarcane *CP4-EPSPS* gene was developed in Argentina. In Argentina, the commercial approval/deregulation of transgenic events requires continuous effort. Extensive research has been conducted on health and environmental issues to evaluate the transgenic event impact on agricultural systems and food safety [27,32,136]. Later, the Ministry of Agriculture, Livestock, and Fisheries denied grants for final approval and commercialization. This may concern the export of sugars produced from such transgenic crops. Nonetheless, this process has led to the development of new regulations for the cultivation of sugarcane. Argentina’s regulatory system for assessing transgenic crops has served as a model in many other countries. In 2013, a significant change was made in the requirements for evaluating vegetatively propagated and highly polyploid genome crops such as sugarcane. This change allowed for fast-track evaluation of new events containing gene constructs equal or similar to those that have already been approved. This resolution has greatly accelerated the approval of new sugarcane varieties with the same or similar gene constructs regardless of their position in the genome. The introgression of a transgene through backcrossing is impossible. Thus, two possible ways to produce transgenic sugarcane varieties have emerged: forward breeding and the direct transformation of elite varieties. Both methods are time-consuming and often result in outdated technology by the time the transgenic variety is ready for commercial release. This policy has allowed the production of new varieties with desirable traits in a timely and cost-effective manner and has been adopted by regulatory agencies in Canada, the United States, and Brazil (Table 5). However, regulatory authorities in various countries play crucial roles in monitoring and overseeing the research and development of GMOs to ensure that their introduction does not pose any threat to human health or the environment. Moreover, due to deregulation, the cultivation and use of transgenic varieties are no longer subject to regulatory restrictions. Therefore, it is necessary to establish stewardship programs to safeguard these varieties and neighboring crops. Implementing current strategies would not only facilitate the successful reintroduction of GM sugarcane but also establish standards for introducing new transgenic traits into sugarcane.

Moreover, the first drought-tolerant transgenic sugarcane expressing the bacterial choline dehydrogenase gene, the osmoregulator glycine betaine, plays a crucial role in plant defenses against cellular dehydration. Persero (PT Perkebunan Nusantara XI) was commercially released in Indonesia (ISAAA 2024; Table 5). Two genetically modified sugarcane varieties produced by Embrapa Agro-energy were approved by the Brazilian regulatory authority, the National Biosafety Technical Commission (CTNBio). Brazilian science has developed the first sugarcane variety with CRISPR/Cas9 altered, Cana Flex I and II, which showed improved cell-wall digestibility. Currently, plants with altered genomes are controlled as transgenic in certain nations but are not regarded as GM in others. Recently, Brazilian authorization has been encouraging as it will significantly reduce the expenses and work required for commercial releases. It is anticipated that more nations will soon assist in the deregulation of varieties created using transgene technology.

## 6. Genome Editing for Sugarcane Improvement

**Figure 4:**
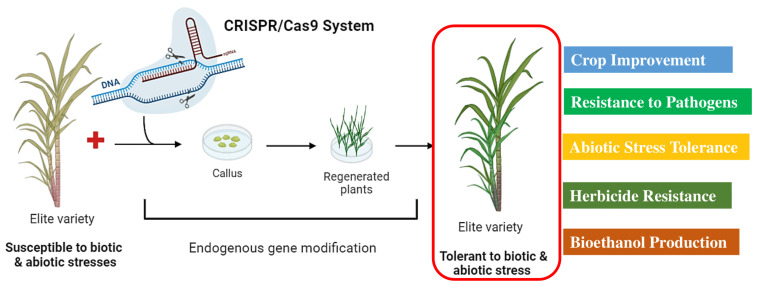
A general description of the genome editing in sugarcane plants to produce elite varieties against biotic and abiotic stress tolerance (https://biorender.com; accessed on 23 April 2024).

Sugarcane is characterized by a huge genome size (~10 GB), high degree of complexity, auto-allopolyploid, limited genetic diversity, and extensive recombination between the two subgenomes. In addition, it is highly heterozygous in nature, taking a long generation time to propagate vegetatively and preserve its genetic makeup. However, alternative strategies such as genetic transformation and the application of different omics technologies have been widely employed for the genetic improvement of sugarcane [137]. Nonetheless, sugarcane can be edited using the CRISPR/Cas9 system, even at high levels of ploidy. CRISPR/Cas9 is a highly effective and dependable tool for targeted mutagenesis (Figure 4). To date, four classes of nuclease-mediated genome editing methods/technologies have been developed; meganucleases, zinc finger nucleases (ZFN), transcription activator-like effector nucleases (TALENs), and the clustered regularly interspaced short palindromic repeats (CRISPR)/Cas9 system [138,139].

Targeted mutagenesis using TALENs was first demonstrated in the sugarcane gene responsible for lignin biosynthesis, caffeic acid O-methyltransferase (*COMT*). The gene was identified, and TALEN-mediated mutations were introduced into the *COMT* gene to assess effects on agronomic performance, lignin content, cell wall composition, and saccharification efficiency. These results showed a 19.7% reduction in lignin and a 44% increase in saccharification of cell wall-bound sugars, without influencing biomass, disease resistance, and logging under field conditions. These modifications significantly increased the production of sugarcane-derived biofuels [130,131]. Furthermore, targeted co-mutagenesis of more than 100 alleles of the *COMT* gene improves saccharification and enhances bioethanol yield from biomass without affecting agronomic performance [138,140]. However, optimizing SgRNA expression cassettes, vectors, and delivery systems is necessary for co-editing multiple alleles. Although TALEN and ZFN-based genome engineering of crops have proven useful, their application for precise gene editing has been limited because of the design and complex interactions of zinc fingers and DNA [138,141]. Of note, sugarcane is a vegetative propagating crop once mutagenesis occurs, and can be stably transmitted to progeny.

The CRISPR/Cas9 system is a groundbreaking technology that emerged as the most crucial tool for crop improvement following genetic transformation [142,143,144]. Among three types of CRISPR/Cas9 mechanisms, Types I, II, and III were classified based on the presence of cas 3, cas 9, and cas 10 genes, respectively; Type II is preferred for genome editing. Using this technology, plants classified under the non-GMO category can be produced, which boosts public acceptance as the target genome does not contain any foreign DNA molecules [145]. Insect pests and diseases are the major causes of reduced sugarcane yield; also, ratoon crops should be considered. Red rot is a major disease that results in significant yield loss in sugarcane. For example, the antifungal diene compound (AFD) is a potent antifungal agent produced in sugarcane that helps to alter dormancy in *Colletotrichum* spp. CRISPR/Cas9 technology can be utilized to modify genes involved in AFD biosynthesis, providing sugarcane resistance to red rot caused by *Colletotrichum* spp. [146].

Additionally, smut disease caused by *S. scitamineum* can be managed by modifying the genes involved in *β*-*1*,*3*-*glucanase* biosynthesis using CRISPR/Cas9. Deletion of *Sspep1* plays a crucial role in various biological activities of *S. scitamineum*, such as mating, defense mechanisms, and virulence, and is helpful in smut disease resistance [147]. Furthermore, deletion of the *Ssubc2* gene encoding kinase regulator in *S. scitamineum* plays a potential role in the growth and proliferation of fungi in sugarcane, resulting in disruption of mating and a decrease in the virulence potential of the fungus [148,149]. Deletion of the *SsRSS1*, which regulates salicylic acid, can result in a reduction in fungal virulence. For instance, R genes play a crucial role in determining the capacity of plants to resist insect pests and diseases, while susceptible genes (*S* genes) make plants sensitive to biotic stress [150]. The adoption of CRISPR/Cas9-mediated genome editing in sugarcane offers several benefits including multiplex capacity, adaptability, and ease of design. This approach is considered more advantageous than the TALEN [151].

CRISPR/Cas9 technology has the potential to improve the expression of the non-specific lipid transfer (*ScNsLTP*) gene in sugarcane, thereby providing tolerance to drought and cold stress [152]. The transcripts of *ScGluD2*, a novel member of subfamily D beta-1,3-glucanase, partially increased after 12 h of salt stress and were significantly upregulated from 6 to 24 h under abscisic acid (ABA), H_2_O_2_, and CdCl_2_. These results suggest that ABA may act as a signaling molecule that helps to regulate oxidative stress and plays a critical role in stress-induced *ScGluD2* transcripts [153]. Interestingly, *ScGluD2* is a stress-responsive gene in sugarcane expressed under defense response against smut, salt, and heavy metal stress [154]. Using CRISPR/Cas9 technology, *ScGluD2* expression can be enhanced to provide tolerance against heavy metal and saline stress in sugarcane.

The use of specific co-editing techniques involving multiple alleles and CRISPR/Cas9 technology can facilitate the rapid development of herbicide tolerance in sugarcane. Analysis of 146 individually engineered plants from five separate experiments in sugarcane revealed targeted nucleotide substitutions, resulting in alterations in W574L and S653I in the acetolactate synthase (*ALS*) gene in 11 lines. In addition to single targeted amino acid substitutions, W574L and S653I were present in 25 and 18 lines, respectively. Co-editing of three *ALS* alleles that confer herbicide tolerance has been established [33], and such techniques can be used to convert inferior to superior alleles through precise substitution mutations.

The application of TALEN-based technology enabled precise editing of conserved locations on the gene, resulting in the development of *COMT*-engineered lines that exhibited a 19.7% reduction in lignin content and a 43.8% improvement in saccharification. The lignin composition of *COMT* mutants increases from 29 to 32% [130]. Considering this fact, and the complex and polyploid nature of the sugarcane genome, precise mutation using CRISPR/Cas9 technology could serve as a valuable tool for reducing lignin content. The utilization of a multigene expression/suppression strategy resulted in the hyper-accumulation of triacylglycerol (TAG) and total fatty acids (FA) in the leaves, as two-fold higher than observed in unmodified energy cane [155,156]. The strongest link between TAG accumulation, *ZmDGAT1* expression, and *SDP1* repression was also observed. Moreover, genetically modified sugarcane upregulates TAG biosynthesis genes and downregulates TAG catabolism genes to direct carbon flux toward oil synthesis and accumulation in the leaf and stem tissues. Additionally, under field conditions, constitutive expression of *WRI 1*, along with lipogenic factors *DGAT1-2*, *OLE1*, and *PXA1*, resulted in hyper-accumulation of TAG and reduced biomass yield [157,158].

## 7. Future Perspectives and Conclusions

Sugarcane is a valuable cash crop that not only provides sugar but also serves as a renewable energy source. The application of genetic engineering and genome editing tools can reduce losses caused by biotic and abiotic stresses. Several transgenic sugarcane traits outperformed under greenhouse and field conditions. However, the commercial release of transgenic sugarcane varieties lags behind that of other crops, such as soybean, corn, and cotton. Nonetheless, some countries, including Argentina, recently allowed the rapid release of second varieties of sugarcane, with genetic modifications similar to previously released varieties. This action is expected to encourage the development of new transgenic varieties in other countries, such as India, China, Thailand, and the United States. With the continuous increase in the world population, excellent transgenic sugarcane varieties such as those with insect resistance, disease resistance, and drought tolerance will attract increasing attention from various countries, thus promoting sucrose production to meet people’s demands. Recent marketing approvals for transgenic sugarcane in Indonesia and Brazil are expected to accelerate the development of new transgenic sugarcane varieties that can provide effective solutions to the challenges faced by crops in different agro-ecological conditions. These improved sugarcane varieties are most urgently needed in Yunnan province, China, because of its huge mountains and extremely complex ecological environment compared to elsewhere in the world. At the same time, advanced biotechnological methods can be used to produce r-proteins, biomolecules, and industrial and pharmaceutical products from the stem vacuoles of sugarcane. Sugarcane can also serve as a platform to produce edible vaccines and other useful products, making plant-based production cost-effective. The successful implementation of sugarcane-based vaccine production could significantly reduce global demand; with the potential to fuel the industry, stimulate economic growth, and reduce carbon emissions, sugarcane is poised to be a game changer.

The CRISPR-Cas9 gene editing method has shown promising results in rice and wheat. However, identifying appropriate sequences for modification and predicting the potential consequences on the genome is a challenging task in sugarcane. However, genome editing in sugarcane has been hindered by transgene silencing at pre–post transcriptional levels. To address this, efficient promoters can be used to regulate specific genome-editing tools, such as Cas9. Like other genome-editing nucleases, Cas9 may also have off-target effects, resulting in unwanted mutations. The gRNA–Cas9 complexes, in addition to cutting target DNA, can also cleave off-target DNA sequences. Overall, several challenges still need to be addressed. The use of different Cas9 variants and other CRISPR-associated nucleases has the potential to be a powerful tool for successful genome editing in sugarcane in the near future.

Furthermore, to effectively utilize CRISPR/Cas9 technology for defending biotic and abiotic stress in sugarcane, a comprehensive understanding of the genomic sequences is indispensable. Fortunately, recent advances in genome sequencing technology and the availability of the reference genome, along with other transcriptome resources, can significantly enhance sugarcane genetic improvement. The CRISPR-Cas9 technology can efficiently edit multiple copies of sugarcane genes after knowing haplotypes clearly, including those with a high number of homologs and homeologs (orthologous and paralogous). Optimizing genome editing reagents and their delivery can facilitate the co-editing of numerous alleles, thereby increasing editing efficiency. Recent achievements in targeting multiple copies of genes in sugarcane have demonstrated that genome editing is possible for polyploid crops. Thus, this opens new avenues for future research to develop new elite varieties with enhanced tolerance to biotic and abiotic stressors. The utilization of gene editing technology is expected to increase soon due to its potential to significantly reduce the time and costs associated with commercial deregulation.

## Figures and Tables

**Table 1:** Genetic modifications for herbicide-tolerant traits in sugarcane.

Variety	Trait Method	Explant	Promoter	Gene	Reference
SP80–180	Particle bombardment	Axillary bud	Ubi-1	*bar*	[20]
ROC22	*Agrobacterium* mediated	Embryogenic calli	Ubi-1	*EPSPS*, *Cry1Ab*	[21]
CPF-234, CPF-213, HSF-240 & CPF-246	Particle bombardment	Embryogenic calli	CaMV35S	*Glyphosate*	[22]
TUC 03-12	Particle bombardment	Embryogenic calli	Rice Actin	*EPSPS*	[23]
CPF-246	*Agrobacterium* mediated	Embryogenic calli	Ubi-1	*Cry1Ac*, *Cry2* & *GT*	[24]
ROC22	*Agrobacterium* mediated	Embryogenic calli	CaMV35S	*bar*	[25]
ROC22	*Agrobacterium* mediated	Embryogenic calli	Ubi-1	*bar*	[26]
RA87-3	Particle bombardment	Embryogenic calli	Rice Actin	*EPSPS*	[27]
NCo310	Particle bombardment	Meristem		*Pat*	[28]
Ja60-15	*Agrobacterium* mediated	Embryogenic calli	Rice-Ubi	*bar*	[29]
Co92061 and Co671	*Agrobacterium* mediated	Somatic embryos	CaMV35S	*bar*	[30]

**Table 2:** List of genetic engineering traits for disease tolerance in sugarcane.

Target Disease	Variety	Trait Method	Explant	Promoter	Gene	Reference
SCMV	SPF-234 & NSG-311	Particle bombardment	Calli	Ubi	*CP*	[1]
SrMV	CP65-357 & CP72-1210	Particle bombardment	Calli	Ubi-1	*CP*	[43]
FDV	Q124	Particle bombardment	Calli	Ubi	Segment 9 of ORF 1	[44]
SCYLV	H62-4671	Particle bombardment	Cell cultures	Ubi	*CP*	[45]
SCMV	Bululawang	*Agrobacterium* mediated	Shoots	CaMV35S	*CP*	[46]
SrMV	ROC22	*Agrobacterium* mediated	Leaf	CaMV35S	*CP*	[47]
SCMV	CP 84-1198 and CP 80-1827	Particle bombardment	Calli	Ubi	*CP*	[48]
SCYLV	CP 92-1666	Particle bombardment	Calli	Ubi	*CP*	[49]
SCMV	Bululawang	PDR & RNAi	Lateral buds		*CP*	[50]
SCSMV	ROC22	*Agrobacterium* mediated	Calli	Ubi 1	*Pac1*	[51]
Red rot	CoJ 83	*Agrobacterium* mediated	Axillary bud	CaMV35S	*β*-*1*,*3*-*glucanase*	[52]
Red rot	S2006SP-93	Particle bombardment	Calli	Ubi	*Chitinase class-II*	[53]
*Colletotrichum*	SPF-234	Particle bombardment	Calli	Ubi	*SWO*, *SWT*	[54]
Smut	KRFo93-1	Particle bombardment	Calli	CaMV35S	*BSR1*	[55]
Leaf scald	Q63 & Q87	Particle bombardment	Calli	Ubi	Albicidin detoxifying	[56]

**Table 3:** List of transgenic sugarcane expressing foreign genes for enhanced insect and pest resistance.

Variety	Trait Method	Explant	Promoter	Candidate Gene	Target Pest	Reference
Ja60-5	Electroporation	Calli	CaMV35S	*cry1Ab*	*D. saccharalis*	[15]
TUC95-10/TUC03-12	Particle bombardment	Calli	pCab1	*Bt*	*Diatraea saccharalis.*	[18]
SP80-1842 & SP80-3280	Particle bombardment	Calli	Maize Ubi-1	*skti, sbbi*	*D. saccharalis*	[20]
ROC22	*Agrobacterium* mediated	Calli	Ubi-1	*cry1Ab*	*D. saccharalis*	[21]
YT79-177 & ROC16	Particle bombardment	Calli	Maize Ubi-1	*cry1Ac*	*P. venosatus*	[64]
CP65-357	Paint-sprayer delivery		Maize Ubi-1	*Snowdrop lectin*	*Eoreuma loftini, D. saccharalis*	[69]
ROC25	*Agrobacterium* mediated	Calli	Ubi-1	*Amaranthus viridis & skti*	*D. saccharalis*	[70]
CoC92061 & Co 86032	Particle bombardment	Calli	Maize Ubi-1	*Aprotinin*	*S. excerptalis*	[71]
CPF-246	*Agrobacterium* mediated	Calli	Maize Ubi-1	*Vip3A*	*C. infuscatellus*	[72]
GT54-9(C9)	*Agrobacterium* mediated	Leaves	CaMV 35S	*cry1Ac*	*Sesamia cretica*	[73]
SP 803280	*Agrobacterium* mediated	Calli	35S & FMV	*cry1Ab, cry2Ab*	*D. saccharalis*	[74]
Zhongzhe1 (ZZ1)	Particle bombardment	Direct embryo	Ubi	PPA	Ceratovacuna lanigera Zehntner	[75]
SP80-3280 & 1842	Particle bombardment	Calli	Maize PEPC	*cry1Ab*	*D. saccharalis*	[76]
YT79-177 & ROC16	Particle bombardment	Calli	Maize Ubi-1	*Synthetic-cry1Ac*	*P. venosatus*	[77]
FN81–745 & Badila	*Agrobacterium* mediated	Calli	RSs-1,Ubi-1	*Gna*	*Ceratovacuna lanigera*	[78]
Gui94-119	Particle bombardment	Calli	Ubi	*cry1Ac*	*D. saccharalis*	[79]
SP80-185	Plasmid transformation	Calli	Maize ubi-1	*HIS Cane CPI -1*	*S. levis*	[80]
CoC671	*Agrobacterium* mediated	Leaf roll	CaMV35S	*cry1Aa3*	*C. infuscatellus, C. sacchariphagu &* *S. excerptalis*	[81]
Co 86032 & CoJ 64	Particle bombardment	Calli	Maize Ubi-1	*cry1Ab*	*C. infuscatellus*	[82]
FN15	Particle bombardment	Calli	CaMV35S	*cry1Ac*	*D. saccharalis*	[83]
LK 92-11	*Agrobacterium* mediated	Calli	CaMV35S	*cry1Ab*	*D. saccharalis*	[84]
SP80-185	Particle bombardment	Calli	Maize ubi-1	*CaneCPI-1*	*S. levis*	[85]
FN15 & ROC22	Particle bombardment	Calli	CaMV35S	*cry1Ac*	*D. saccharalis*	[86]
ROC22	Particle bombardment	Calli	ST-LSI	*cry2A*	*C. sacchariphagus,* *S. nivella, C. infuscatellus, A. schistaceana &* *S. inferens*	[87]
Event CTC175-A	*Agrobacterium* mediated	Calli	PEPC	*cry1Ab*	*D. saccharalis*	[88]
Event CTC91087-6	*Agrobacterium* mediated	Calli	Maize ubi-1	*Cry1ac*	*D. saccharalis*	[89]
Bululawang	*Agrobacterium* mediated	Calli	RUBISCO	*CryIAb-CryIAc*	*Scripophaga excerptalis*	[90]

**Table 4:** Genetic engineering of sugarcane for abiotic stress tolerance.

Variety	Trait Method	Type of Explant	Promoter	Gene	Gene Function	Stress	Reference
ROC22	*Agrobacterium*		CaMV35S	*TERF1*	Gene regulation	Drought	[2]
RB855156	Biolistic	Embryogenic calli	pRab17	*DREB2A CA*	Gene regulation	Drought	[100]
NCo310	Biolistic	Embryogenic calli	UBI	*AtBBX29*	Gene regulation	Drought	[101]
CP-77-400	*Agrobacterium*	Apical buds	CaMV35S	*AVP1*	Osmotic regulation	Drought	[102]
CSSG-668	Biolistic	Embryogenic calli	CaMV35S	*AVP1*	Osmotic regulation	Drought	[103]
RB835089	Biolistic	Embryogeniccalli	UBI	*BI-1*	PCD-regulation	Drought	[104]
RB855156	Biolistic	Nodal buds	ABA-AIPC	*P5CS*	Proline synthesis	Salinity	[105]
Guitang21	*Agrobacterium*	Embryogenic calli	UBI	*SoP5CS*	Proline synthesis	Drought	[106]
Co 86032	Biolistic		UBI	*EaGly III*	Reduce oxidative stress	Salinity	[107]
Co86032	*Agrobacterium*		UBI	*HSP70*	Cellular stability	Drought/ Salinity	[108]
RB855536	Biolistic	Embryogenic calli	AtCOR15a	*ipt*	Cytokinin	Cold	[109]
ROC22	*Agrobacterium*	Calli	UBI	*SoTUA*	α-tubulin synthesis	Cold	[110]
ROC10	*Agrobacterium*		CaMV35S	*TSase*	Biomolecules stabilization	Drought	[111]
Co86032	*Agrobacterium*/ biobalistic	Embryogenic calli	UBI	*PDH45/DREB2*	Nucleic acids metabolism	Drought/ Salinity	[112]

**Table 5:** List of Sugarcane genetically modified (GM) event names approved for food/feed and commercialization.

Developer	Event Name	Trait Method	Gene	Gene Source	Product	Function	Country/Year
Centro de Tecnologia Canavieira (CTC)	CTB141175/01-A	Microparticle bombardment	*cry1Ab*	*B. thuringiensis* subsp. kurstaki	Cry1Ab delta-endotoxin	lepidopteron insects	Brazil 2017, Canada & United States 2018
	CTC-92015-7	*Agrobacterium* mediated	*cry1Ac*	*B. thuringiensis* subsp. Kurstaki strain HD73	Cry1Ac delta-endotoxin	lepidopteron insects	Brazil 2022
			*nptII*	*E.coli* Tn5 transposon	neomycin phosphotransferase II	neomycin & kanamycin antibiotics	Brazil 2022
	CTC75064-3	*Agrobacterium* mediated	*cry1Ac*	*B.thuringiensis* subsp. Kurstaki strain HD73	Cry1Ac delta-endotoxin	lepidopteron insects	Brazil 2020, Canada 2022
			*nptII*	*E.coli* Tn5 transposon	neomycin phosphotransferase II	neomycin & kanamycin antibiotics	Brazil 2020, Canada 2022
	CTC91087-6		*cry1Ac*	*B. thuringiensis* subsp. Kurstaki strain HD73	Cry1Ac delta-endotoxin	lepidopteron insects	Brazil 2018 & United States 2020
	CTC93209	*Agrobacterium* mediated	*cry1Ac*	*B. thuringiensis* subsp. Kurstaki strain HD73	Cry1Ac delta-endotoxin	lepidopteron insects	Brazil 2019
	CTC95019-5	*Agrobacterium* mediated	*cry1Ac*	*B. thuringiensis* subsp. Kurstaki strain HD73	Cry1Ac delta-endotoxin	lepidopteron insects	Brazil 2021
			*nptII*	*E. coli* Tn5 transposon	neomycin phospho transferase II	neomycin & kanamycin antibiotics	Brazil 2021
PT Perkebunan Nusantara XI (Persero)	NXI-1T	*Agrobacterium* mediated	*EcBetA*	*E. coli*	choline dehydrogenase	Osmoprotectant, glycine betaine	Indonesia 2011
			*nptII*	*E. coli* Tn5 transposon	neomycin phospho transferase II	neomycin & kanamycin	Indonesia 2011
			*aph4 hpt)*	*E. coli*	hygromycin-B phosphotransferase	hygromycin B	Indonesia 2011
PT Perkebunan Nusantara XI (Persero)	NXI-4T	*Agrobacterium* mediated	*RmBetA*	*Rhizobium meliloti*	choline dehydrogenase	osmoprotectantglycine betaine)	Indonesia 2013
PT Perkebunan Nusantara XI (Persero)	NXI-6T	*Agrobacterium* mediated	*RmBetA*	*Rhizobium meliloti*	choline dehydrogenase	Osmoprotectant, glycine betaine	Indonesia 2013
Estación Experimental Agroindustrial Obispo Colombres (EEAOC)	TUC-873RH-7	Microparticle bombardment	*cp4 epsps (aroA:CP4)*	*A. tumefaciens* strain CP4	(EPSPS) enzyme	glyphosate herbicide	Argentina 2015
			*nptII*	*E. coli* Tn5 transposon	neomycin phosphotransferase II	neomycin & kanamycin antibiotics	Argentina 2015
Monsanto Company & Bayer Crop Science	MON87427 × MON95379 × MON87411	Conventional breeding-cross hybridization-transgenic donor(s)	*cry1Da_7*	*B. thuringiensis*	crystalline protein prototoxin Cry1Da_7	*S. italica* promoter and O. sativa gos2 terminator-Rice actin 15 gene-intron	Brazil 2021
			*cry1B.868*	*B. thuringiensis*	crystalline protein prototoxin *Cry1B*.868	*S. italica* promoter and O. sativa gos2 terminator-Rice actin 15 gene-intron	Brazil 2021
			*cry3Bb1*	*B. thuringiensis* subsp. kumamotoensis	*Cry3Bb1* delta endotoxin	Tolerance to coleopteran insects & corn rootworm	Brazil 2021

Source 2024. Crop Biotech Update April. Available online at: https://www.isaaa.org/gmapprovaldatabase/crop/default.asp? CropID=27&Crop=Sugarcane (accessed on 24 April 2024).

## References

[1] Aslam U., Tabassum B., Nasir I.A., Khan A., Husnain T. (2018). A Virus-Derived Short Hairpin RNA Confers Resistance against Sugarcane Mosaic Virus in Transgenic Sugarcane. Transgenic Res..

[2] Rahman M.A., Wu W., Yan Y., Bhuiyan S.A. (2021). Overexpression of *TERF1* in Sugarcane Improves Tolerance to Drought Stress. Crop Pasture Sci..

[3] Grandis A., Fortirer J.S., Navarro B.V., de Oliveira L.P., Buckeridge M.S. (2023). Biotechnologies to Improve Sugarcane Productivity in a Climate Change Scenario. BioEnergy Res..

[4] Piperidis N., D’Hont A. (2020). Sugarcane Genome Architecture Decrypted with Chromosome-Specific Oligo Probes. Plant J..

[5] Vieira M.L.C., Almeida C.B., Oliveira C.A., Tacuatiá L.O., Munhoz C.F., Cauz-Santos L.A., Pinto L.R., Monteiro-Vitorello C.B., Xavier M.A., Forni-Martins E.R. (2018). Revisiting Meiosis in Sugarcane: Chromosomal Irregularities and the Prevalence of Bivalent Configurations. Front. Genet..

[6] Zhang J., Zhang X., Tang H., Zhang Q., Hua X., Ma X., Zhu F., Jones T., Zhu X., Bowers J. (2018). Allele-Defined Genome of the Autopolyploid Sugarcane *Saccharum spontaneum* L. Nat. Genet..

[7] Garsmeur O., Droc G., Antonise R., Grimwood J., Potier B., Aitken K., Jenkins J., Martin G., Charron C., Hervouet C. (2018). A Mosaic Monoploid Reference Sequence for the Highly Complex Genome of Sugarcane. Nat. Commun..

[8] Souza G.M., Van Sluys M.-A., Lembke C.G., Lee H., Margarido G.R.A., Hotta C.T., Gaiarsa J.W., Diniz A.L., Oliveira M.d.M., Ferreira S.d.S. (2019). Assembly of the 373k Gene Space of the Polyploid Sugarcane Genome Reveals Reservoirs of Functional Diversity in the World’s Leading Biomass Crop. Gigascience.

[9] Zhang Q., Qi Y., Pan H., Tang H., Wang G., Hua X., Wang Y., Lin L., Li Z., Li Y. (2022). Genomic Insights into the Recent Chromosome Reduction of Autopolyploid Sugarcane *Saccharum spontaneum*. Nat. Genet..

[10] Wang T., Wang B., Hua X., Tang H., Zhang Z., Gao R., Qi Y., Zhang Q., Wang G., Yu Z. (2023). A Complete Gap-Free Diploid Genome in *Saccharum* Complex and the Genomic Footprints of Evolution in the Highly Polyploid *Saccharum* Genus. Nat. Plants.

[11] Kui L., Majeed A., Wang X., Yang Z., Chen J., He L., Di Y., Li X., Qian Z., Jiao Y. (2023). A Chromosome-Level Genome Assembly for *Erianthus fulvus* Provides Insights into Its Biofuel Potential and Facilitates Breeding for Improvement of Sugarcane. Plant Commun..

[12] Bao Y., Zhang Q., Huang J., Zhang S., Yao W., Yu Z., Deng Z., Yu J., Kong W., Yu X. (2024). A Chromosomal-Scale Genome Assembly of Modern Cultivated Hybrid Sugarcane Provides Insights into Origination and Evolution. Nat. Commun..

[13] Healey A.L., Garsmeur O., Lovell J.T., Shengquiang S., Sreedasyam A., Jenkins J., Plott C.B., Piperidis N., Pompidor N., Llaca V. (2024). The Complex Polyploid Genome Architecture of Sugarcane. Nature.

[14] Bower R., Birch R.G. (1992). Transgenic Sugarcane Plants via Microprojectile Bombardment. Plant J..

[15] Arencibia A.D., Carmona E.R., Tellez P., Chan M.-T., Yu S.-M., Trujillo L.E., Oramas P. (1998). An Efficient Protocol for Sugarcane (*Saccharum* spp. L.) Transformation Mediated by *Agrobacterium tumefaciens*. Transgenic Res..

[16] Budeguer F., Enrique R., Perera M.F., Racedo J., Castagnaro A.P., Noguera A.S., Welin B. (2021). Genetic Transformation of Sugarcane, Current Status and Future Prospects. Front. Plant Sci..

[17] Li A.-M., Chen Z.-L., Liao F., Zhao Y., Qin C.-X., Wang M., Pan Y.-Q., Wei S.-L., Huang D.-L. (2024). Sugarcane Borers: Species, Distribution, Damage and Management Options. J. Pest. Sci..

[18] Budeguer F., Racedo J., Enrique R., Perera M.F., Ostengo S., Noguera A.S. (2024). Transgenic Sugarcane for the Sustainable Management of the Sugarcane Borer *Diatraea saccharalis*. Sugar Ind..

[19] Mulwa R.M.S., Mwanza L.M. (2006). Review-Biotechnology Approaches to Developing Herbicide Tolerance/Selectivity in Crops. Afr. J. Biotechnol..

[20] Falco M.C., Tulmann Neto A., Ulian E.C. (2000). Transformation and Expression of a Gene for Herbicide Resistance in a Brazilian Sugarcane. Plant Cell Rep..

[21] Wang W.Z., Yang B.P., Feng X.Y., Cao Z.Y., Feng C.L., Wang J.G., Xiong G.R., Shen L.B., Zeng J., Zhao T.T. (2017). Development and Characterization of Transgenic Sugarcane with Insect Resistance and Herbicide Tolerance. Front. Plant Sci..

[22] Nasir I., Tabassum B., Qamar Z., Javed M., Tariq M., Farooq A., Butt S., Qayyum A., Husnain T. (2014). Herbicide-Tolerant Sugarcane (*Saccharum officinarum* L.) Plants: An Unconventional Method of Weed Removal. Turk. J. Biol..

[23] Racedo J., Noguera A.S., Castagnaro A.P., Perera M.F. (2023). Biotechnological Strategies Adopted for Sugarcane Disease Management in Tucumán, Argentina. Plants.

[24] Qamar Z., Nasir I.A., Abouhaidar M.G., Hefferon K.L., Rao A.Q., Latif A., Ali Q., Anwar S., Rashid B., Shahid A.A. (2021). Novel Approaches to Circumvent the Devastating Effects of Pests on Sugarcane. Sci. Rep..

[25] Zan F., Wu Z., Wang W., Hu X., Feng L., Liu X., Liu J., Zhao L., Wu C., Zhang S. (2023). Strigolactones in Sugarcane Growth and Development. Agronomy.

[26] Wang W., Javed T., Shen L., Sun T., Yang B., Zhang S. (2024). Establishment of an Efficient Sugarcane Transformation System via Herbicide-Resistant *CP4-EPSPS* Gene Selection. Plants.

[27] Noguera A., Enrique R., Ostengo S., Perera M.F., Racedo J., Costilla D., Zossi S., Cuenya M.I., Paula M., Welin B. (2019). Development of the Transgenic Sugarcane Event TUC 87-3RG Resistant to Glyphosate. Proc. Int. Soc. Sugar Cane Technol..

[28] Leibbrandt N.B., Snyman S.J. (2003). Stability of Gene Expression and Agronomic Performance of a Transgenic Herbicide-Resistant Sugarcane Line in South Africa. Crop Sci..

[29] Enríquez-Obregón G.A., Vázquez-Padrón R.I., Prieto-Samsonov D.L., De la Riva G.A., Selman-Housein G. (1998). Herbicide-Resistant Sugarcane (*Saccharum officinarum* L.) Plants by *Agrobacterium*-Mediated Transformation. Planta.

[30] Manickavasagam M., Ganapathi A., Anbazhagan V.R., Sudhakar B., Selvaraj N., Vasudevan A., Kasthurirengan S. (2004). *Agrobacterium*-Mediated Genetic Transformation and Development of Herbicide-Resistant Sugarcane (*Saccharum* Species Hybrids) Using Axillary Buds. Plant Cell Rep..

[31] Wang W.Z., Yang B.P., Feng C.L., Wang J.G., Xiong G.R., Zhao T.T., Zhang S.Z. (2017). Efficient Sugarcane Transformation via Bar Gene Selection. Trop. Plant Biol..

[32] Ostengo S., Serino G., Perera M.F., Racedo J., Mamaní González S.Y., Yáñez Cornejo F., Cuenya M.I. (2022). Sugarcane Breeding, Germplasm Development and Supporting Genetic Research in Argentina. Sugar Tech.

[33] Oz M.T., Altpeter A., Karan R., Merotto A., Altpeter F. (2021). CRISPR/Cas9-mediated Multi-Allelic Gene Targeting in Sugarcane Confers Herbicide Tolerance. Front. Genome Ed..

[34] Souza T.P., Dias R.O., Silva-Filho M.C. (2017). Defense-Related Proteins Involved in Sugarcane Responses to Biotic Stress. Genet. Mol. Biol..

[35] Chu N., Zhou J.-R., Rott P.C., Li J., Fu H.-Y., Huang M.-T., Zhang H.-L., Gao S.-J. (2022). *ScPR1* Plays a Positive Role in the Regulation of Resistance to Diverse Stresses in Sugarcane (*Saccharum* spp.) and *Arabidopsis thaliana*. Ind. Crops Prod..

[36] Zhou J.-R., Sun H.-D., Ali A., Rott P.C., Javed T., Fu H.-Y., Gao S.-J. (2021). Quantitative Proteomic Analysis of the Sugarcane Defense Responses Incited by *Acidovorax avenae* subsp. avenae Causing Red Stripe. Ind. Crops Prod..

[37] Javed T., Gao S.-J. (2023). *WRKY* Transcription Factors in Plant Defense. Trends Genet..

[38] Javed T., Shabbir R., Ali A., Afzal I., Zaheer U., Gao S.-J. (2020). Transcription Factors in Plant Stress Responses: Challenges and Potential for Sugarcane Improvement. Plants.

[39] Wang Y., Zhang J., Wang R., Hou Y., Fu H., Xie Y., Gao S., Wang J. (2021). Unveiling Sugarcane Defense Response to *Mythimna separata* Herbivory by a Combination of Transcriptome and Metabolic Analyses. Pest. Manag. Sci..

[40] Hu Z.-T., Ntambo M.S., Zhao J.-Y., Javed T., Shi Y., Fu H.-Y., Huang M.-T., Gao S.-J. (2023). Genetic Divergence and Population Structure of *Xanthomonas albilineans* Strains Infecting *Saccharum* spp. Hybrid and *Saccharum officinarum*. Plants.

[41] Verma K.K., Song X.-P., Budeguer F., Nikpay A., Enrique R., Singh M., Zhang B.-Q., Wu J.-M., Li Y.-R. (2022). Genetic Engineering: An Efficient Approach to Mitigating Biotic and Abiotic Stresses in Sugarcane Cultivation. Plant Signal. Behav..

[42] Cursi D.E., Castillo R.O., Tarumoto Y., Umeda M., Tippayawat A., Ponragdee W., Racedo J., Perera M.F., Hoffmann H.P., Carneiro M.S., Priyadarshan P.M., Jain S.M. (2022). Origin, Genetic Diversity, Conservation, and Traditional and Molecular Breeding Approaches in Sugarcane. Cash Crops: Genetic Diversity, Erosion, Conservation and Utilization.

[43] Ingelbrecht I.L., Irvine J.E., Mirkov T.E. (1999). Posttranscriptional Gene Silencing in Transgenic Sugarcane. Dissection of Homology-Dependent Virus Resistance in a Monocot That Has a Complex Polyploid Genome. Plant Physiol..

[44] McQualter R.B., Dale J.L., Harding R.M., McMahon J.A., Smith G.R. (2004). Production and Evaluation of Transgenic Sugarcane Containing a Fiji Disease Virus (FDV) Genome Segment S9-Derived Synthetic Resistance Gene. Aust. J. Agric. Res..

[45] Zhu Y.J., McCafferty H., Osterman G., Lim S., Agbayani R., Lehrer A., Schenck S., Komor E. (2011). Genetic Transformation with Untranslatable Coat Protein Gene of Sugarcane Yellow Leaf Virus Reduces Virus Titers in Sugarcane. Transgenic Res..

[46] Apriasti R., Widyaningrum S., Hidayati W.N., Sawitri W.D., Darsono N., Hase T., Sugiharto B. (2018). Full Sequence of the Coat Protein Gene Is Required for the Induction of Pathogen-Derived Resistance against Sugarcane Mosaic Virus in Transgenic Sugarcane. Mol. Biol. Rep..

[47] Guo J., Gao S., Lin Q., Wang H., Que Y., Xu L. (2015). Transgenic Sugarcane Resistant to *Sorghum Mosaic Virus* Based on Coat Protein Gene Silencing by RNA Interference. BioMed Res. Int..

[48] Gilbert R.A., Gallo-Meagher M., Comstock J.C., Miller J.D., Jain M., Abouzid A. (2005). Agronomic Evaluation of Sugarcane Lines Transformed for Resistance to Sugarcane Mosaic Virus Strain E. Crop Sci..

[49] Gilbert R.A., Glynn N.C., Comstock J.C., Davis M.J. (2009). Agronomic Performance and Genetic Characterization of Sugarcane Transformed for Resistance to Sugarcane Yellow Leaf Virus. Field Crops Res..

[50] Hidayati W.N., Apriasti R., Addy H.S., Sugiharto B. (2021). Distinguishing Resistances of Transgenic Sugarcane Generated from RNA Interference and Pathogen-derived Resistance Approaches to Combating Sugarcane Mosaic Virus. Indones. J. Biotechnol..

[51] Wang W., Wang J., Feng X., Shen L., Feng C., Zhao T., Xiao H., Li S., Zhang S. (2022). Breeding of Virus-Resistant Transgenic Sugarcane by the Integration of the *Pac1* Gene. Front. Sustain. Food Syst..

[52] Nayyar S., Sharma B.K., Kaur A., Kalia A., Sanghera G.S., Thind K.S., Yadav I.S., Sandhu J.S. (2017). Red Rot Resistant Transgenic Sugarcane Developed through Expression of *β*-*1*,*3*-*glucanase* Gene. PLoS ONE.

[53] Tariq M., Khan A., Tabassum B., Toufiq N., Bhatti M.U., Riaz S., Nasir I.A., Husnain T. (2018). Antifungal Activity of Chitinase II against *Colletotrichum falcatum* Went. Causing Red Rot Disease in Transgenic Sugarcane. Turk. J. Biol..

[54] Parvaiz A., Mustafa G., Khan M.S., Ali M.A. (2021). Over-Expression of Endogenous *SUGARWIN* Genes Exalted Tolerance against *Colletotrichum* Infection in Sugarcane. Plants.

[55] Maeda S., Ackley W., Yokotani N., Sasaki K., Ohtsubo N., Oda K., Mori M. (2023). Enhanced Resistance to Fungal and Bacterial Diseases due to Overexpression of *BSR1*, a Rice *RLCK*, in Sugarcane, Tomato, and Torenia. Int. J. Mol. Sci..

[56] Zhang L., Xu J., Birch R.G. (1999). Engineered Detoxification Confers Resistance against a Pathogenic Bacterium. Nat. Biotechnol..

[57] Babu K.H., Devarumath R.M., Thorat A.S., Nalavade V.M., Saindane M., Appunu C., Suprasanna P., Kavi Kishor P.B., Rajam M.V., Pullaiah T. (2021). Sugarcane Transgenics: Developments and Opportunities. Genetically Modified Crops: Current Status, Prospects and Challenges Volume 1.

[58] Sawitri W.D., Harmoko R., Sugiharto B. (2024). Induction of Resistance against Sugarcane Mosaic Virus by Pathogen-Derived Resistance and RNA Interference Methods in Transgenic Sugarcane. AIP Conf. Proc..

[59] Viswanathan C., Anburaj J., Prabu G. (2014). Identification and Validation of Sugarcane Streak Mosaic Virus-Encoded microRNAs and Their Targets in Sugarcane. Plant Cell Rep..

[60] Widyaningrum S., Pujiasih D.R., Sholeha W., Harmoko R., Sugiharto B. (2021). Induction of Resistance Tolerance to Sugarcane Mosaic Virus by RNA Interference Targeting Coat Protein Gene Silencing in Transgenic Sugarcane. Mol. Biol. Rep..

[61] Yao W., Ruan M., Qin L., Yang C., Chen R., Chen B., Zhang M. (2017). Field Performance of Transgenic Sugarcane Lines Resistant to Sugarcane Mosaic Virus. Front. Plant Sci..

[62] Noguera A., Enrique R., Perera M.F., Ostengo S., Racedo J., Costilla D., Zossi S., Cuenya M.I., Filippone M.P., Welin B. (2015). Genetic Characterization and Field Evaluation to Recover Parental Phenotype in Transgenic Sugarcane: A Step toward Commercial Release. Mol. Breed..

[63] Nerkar G., Thorat A., Sheelavantmath S., Kassa H.B., Devarumath R., Gosal S.S., Wani S.H. (2018). Genetic Transformation of Sugarcane and Field Performance of Transgenic Sugarcane. Biotechnologies of Crop Improvement, Volume 2: Transgenic Approaches.

[64] Weng L.-X., Deng H.-H., Xu J.-L., Li Q., Zhang Y.-Q., Jiang Z.-D., Li Q.-W., Chen J.-W., Zhang L.-H. (2011). Transgenic Sugarcane Plants Expressing High Levels of Modified *cry1Ac* Provide Effective Control against Stem Borers in Field Trials. Transgenic Res..

[65] Iqbal A., Khan R.S., Khan M.A., Gul K., Jalil F., Shah D.A., Rahman H., Ahmed T. (2021). Genetic Engineering Approaches for Enhanced Insect Pest Resistance in Sugarcane. Mol. Biotechnol..

[66] Schneider V.K., Soares-Costa A., Chakravarthi M., Ribeiro C., Chabregas S.M., Falco M.C., Henrique-Silva F. (2017). Transgenic Sugarcane Overexpressing *CaneCPI*-1 Negatively Affects the Growth and Development of the Sugarcane Weevil *Sphenophorus* levis. Plant Cell Rep..

[67] Gill R., Malhotra P.K., Gosal S.S. (2006). Direct Plant Regeneration from Cultured Young Leaf Segments of Sugarcane. Plant Cell Tissue Organ Cult..

[68] Gosal S.S., Wani S.H., Gosal S.S., Wani S.H. (2018). Plant Genetic Transformation and Transgenic Crops: Methods and Applications. Biotechnologies of Crop Improvement, Volume 2: Transgenic Approaches.

[69] Sétamou M., Bernal J.S., Legaspi J.C., Mirkov T.E., Legaspi B.C. (2002). Evaluation of *Lectin*-Expressing Transgenic Sugarcane against Stalkborers (Lepidoptera: Pyralidae): Effects on Life History Parameters. J. Econ. Entomol..

[70] Deng Z.-N., Wei Y.-W., Lü W.-L., Li Y.-R. (2008). Fusion Insect-Resistant Gene Mediated by Matrix Attachment Region (MAR) Sequence in Transgenic Sugarcane. Sugar Tech.

[71] Christy L.A., Arvinth S., Saravanakumar M., Kanchana M., Mukunthan N., Srikanth J., Thomas G., Subramonian N. (2009). Engineering Sugarcane Cultivars with Bovine Pancreatic Trypsin Inhibitor (*Aprotinin*) Gene for Protection against Top Borer (*Scirpophaga excerptalis* Walker). Plant Cell Rep..

[72] Riaz S., Nasir I.A., Bhatti M.U., Adeyinka O.S., Toufiq N., Yousaf I., Tabassum B. (2020). Resistance to *Chilo infuscatellus* (Lepidoptera: Pyraloidea) in Transgenic Lines of Sugarcane Expressing *Bacillus thuringiensis* Derived Vip3A Protein. Mol. Biol. Rep..

[73] Dessoky E.S., Ismail R.M., Elarabi N.I., Abdelhadi A.A., Abdallah N.A. (2021). Improvement of Sugarcane for Borer Resistance Using *Agrobacterium* Mediated Transformation of *cry1Ac* Gene. GM Crops Food.

[74] Cristofoletti P.T., Kemper E.L., Capella A.N., Carmago S.R., Cazoto J.L., Ferrari F., Galvan T.L., Gauer L., Monge G.A., Nishikawa M.A. (2018). Development of Transgenic Sugarcane Resistant to Sugarcane Borer. Trop. Plant Biol..

[75] Zhao M., Zhou Y., Su L., Li G., Huang Z., Huang D., Wu W., Zhao Y. (2022). Expression of *Pinellia pedatisecta* Agglutinin *PPA* Gene in Transgenic Sugarcane Led to Stomata Patterning Change and Resistance to Sugarcane Woolly Aphid, *Ceratovacuna lanigera* Zehntner. Int. J. Mol. Sci..

[76] Braga D.P.V., Arrigoni E.D.B., Silva-Filho M.C., Ulian E.C. (2003). Expression of the *Cry1Ab* Protein in Genetically Modified Sugarcane for the Control of *Diatraea saccharalis* (Lepidoptera: Crambidae). J. New Seeds.

[77] Weng L.-X., Deng H., Xu J.-L., Li Q., Wang L.-H., Jiang Z., Zhang H.B., Li Q., Zhang L.-H. (2006). Regeneration of Sugarcane Elite Breeding Lines and Engineering of Stem Borer Resistance. Pest. Manag. Sci..

[78] Zhangsun D., Luo S., Chen R., Tang K. (2007). Improved *Agrobacterium*-Mediated Genetic Transformation of *GNA* Transgenic Sugarcane. Biologia.

[79] Xu J.-S., Gao S., Xu L., Chen R. (2008). Construction of Expression Vector of *CryIA(c)* Gene and Its Transformation in Sugarcane. Sugar Tech.

[80] Ribeiro C.W., Soares-Costa A., Falco M.C., Chabregas S.M., Ulian E.C., Cotrin S.S., Carmona A.K., Santana L.A., Oliva M.L.V., Henrique-Silva F. (2008). Production of a His-Tagged Canecystatin in Transgenic Sugarcane and Subsequent Purification. Biotechnol. Prog..

[81] Kalunke R.M., Kolge A.M., Babu K.H., Prasad D.T. (2009). *Agrobacterium* Mediated Transformation of Sugarcane for Borer Resistance Using *Cry 1Aa3* Gene and One-Step Regeneration of Transgenic Plants. Sugar Tech.

[82] Arvinth S., Arun S., Selvakesavan R.K., Srikanth J., Mukunthan N., Ananda Kumar P., Premachandran M.N., Subramonian N. (2010). Genetic Transformation and Pyramiding of Aprotinin-Expressing Sugarcane with *cry1Ab* for Shoot Borer (*Chilo infuscatellus*) Resistance. Plant Cell Rep..

[83] Gao S., Yang Y., Wang C., Guo J., Zhou D., Wu Q., Su Y., Xu L., Que Y. (2016). Transgenic Sugarcane with a *cry1Ac* Gene Exhibited Better Phenotypic Traits and Enhanced Resistance against Sugarcane Borer. PLoS ONE.

[84] Islam N., Laksana C., Chanprame S. (2016). *Agrobacterium*-mediated Transformation and Expression of *Bt* Gene in Transgenic Sugarcane. J. Int. Soc. Southeast Asian Agric. Sci..

[85] Shibao P.Y.T., Santos-Júnior C.D., Santiago A.C., Mohan C., Miguel M.C., Toyama D., Vieira M.A.S., Narayanan S., Figueira A., Carmona A.K. (2021). Sugarcane Cystatins: From Discovery to Biotechnological Applications. Int. J. Biol. Macromol..

[86] Zhou D., Liu X., Gao S., Guo J., Su Y., Ling H., Wang C., Li Z., Xu L., Que Y. (2018). Foreign *cry1Ac* Gene Integration and Endogenous Borer Stress-Related Genes Synergistically Improve Insect Resistance in Sugarcane. BMC Plant Biol..

[87] Gao S., Yang Y., Xu L., Guo J., Su Y., Wu Q., Wang C., Que Y. (2018). Particle Bombardment of the *cry2A* Gene Cassette Induces Stem Borer Resistance in Sugarcane. Int. J. Mol. Sci..

[88] Cheavegatti-Gianotto A., Gentile A., Oldemburgo D.A., Merheb G.d.A., Sereno M.L., Lirette R.P., Ferrseira T.H.S., de Oliveira W.S. (2018). Lack of Detection of *Bt* Sugarcane *Cry1Ab* and *nptII* DNA and Proteins in Sugarcane Processing Products Including Raw Sugar. Front. Bioeng. Biotechnol..

[89] Gianotto A.C., Rocha M.S., Cutri L., Lopes F.C., Dal’Acqua W., Hjelle J.J., Lirette R.P., Oliveira W.S., Sereno M.L. (2019). The Insect-Protected CTC91087-6 Sugarcane Event Expresses *Cry1Ac* Protein Preferentially in Leaves and Presents Compositional Equivalence to Conventional Sugarcane. GM Crops Food.

[90] Koerniati S., Sukmadjaja D., Samudra I.M. (2020). C Synthetic Gene of *CryIAb-CryIAc* Fusion to Generate Resistant Sugarcane to Shoot or Stem Borer. IOP Conf. Ser. Earth Environ. Sci..

[91] Punithavalli M., Jebamalaimary A. (2019). Inhibitory Activities of Proteinase Inhibitors on Developmental Characteristics of Sugarcane *Chilo infuscatellus* (Snellen). Phytoparasitica.

[92] Punithavalli M. (2022). Spatial Distribution of Proteinase Inhibitors among Diverse Groups of Sugarcane and Their Interaction with Sugarcane Borers. Indian J. Entomol..

[93] Salgado L.D., Wilson B.E., Villegas J.M., Richard R.T., Penn H.J. (2022). Resistance to the Sugarcane Borer (Lepidoptera: Crambidae) in Louisiana Sugarcane Cultivars. Environ. Entomol..

[94] Dixit S., Sivalingam P.N., Baskaran R.K.M., Senthil-Kumar M., Ghosh P.K. (2024). Plant Responses to Concurrent Abiotic and Biotic Stress: Unravelling Physiological and Morphological Mechanisms. Plant Physiol. Rep..

[95] Zhang H., Zhu J., Gong Z., Zhu J.-K. (2022). Abiotic Stress Responses in Plants. Nat. Rev. Genet..

[96] Wei Y.-S., Zhao J.-Y., Javed T., Ali A., Huang M.-T., Fu H.-Y., Zhang H.-L., Gao S.-J. (2024). Insights into Reactive Oxygen Species Production-Scavenging System Involved in Sugarcane Response to *Xanthomonas albilineans* Infection under Drought Stress. Plants.

[97] Mishra N., Jiang C., Chen L., Paul A., Chatterjee A., Shen G. (2023). Achieving Abiotic Stress Tolerance in Plants through Antioxidative Defense Mechanisms. Front. Plant Sci..

[98] Tardieu F. (2012). Any Trait or Trait-Related Allele Can Confer Drought Tolerance: Just Design the Right Drought Scenario. J. Exp. Bot..

[99] Cominelli E., Conti L., Tonelli C., Galbiati M. (2013). Challenges and Perspectives to Improve Crop Drought and Salinity Tolerance. New Biotechnol..

[100] Reis R.R., Andrade Dias Brito da Cunha B., Martins P.K., Martins M.T.B., Alekcevetch J.C., Chalfun-Júnior A., Andrade A.C., Ribeiro A.P., Qin F., Mizoi J. (2014). Induced Over-Expression of *AtDREB2A CA* Improves Drought Tolerance in Sugarcane. Plant Sci..

[101] Mbambalala N., Panda S.K., van der Vyver C. (2021). Overexpression of *AtBBX29* Improves Drought Tolerance by Maintaining Photosynthesis and Enhancing the Antioxidant and Osmolyte Capacity of Sugarcane Plants. Plant Mol. Biol. Rep..

[102] Kumar T., Uzma, Khan M.R., Abbas Z., Ali G.M. (2014). Genetic Improvement of Sugarcane for Drought and Salinity Stress Tolerance Using *Arabidopsis* Vacuolar Pyrophosphatase (*AVP1*) Gene. Mol. Biotechnol..

[103] RAZA G., Ali K., Ashraf M., Mansoor S., Javid M., Asad S. (2016). Overexpression of an *H^+^-PPase* Gene from *Arabidopsis* in Sugarcane Improves drought Tolerance, Plant Growth, and Photosynthetic Responses. Turk. J. Biol..

[104] Ramiro D.A., Melotto-Passarin D.M., Barbosa M.d.A., dos Santos F., Gomez S.G.P., Massola Júnior N.S., Lam E., Carrer H. (2016). Expression of Arabidopsis Bax Inhibitor-1 in Transgenic Sugarcane Confers Drought Tolerance. Plant Biotechnol. J..

[105] Guerzoni J.T.S., Belintani N.G., Moreira R.M.P., Hoshino A.A., Domingues D.S., Filho J.C.B., Vieira L.G.E. (2014). Stress-Induced Δ1-Pyrroline-5-Carboxylate Synthetase (*P5CS*) Gene Confers Tolerance to Salt Stress in Transgenic Sugarcane. Acta Physiol. Plant..

[106] Li J., Phan T.-T., Li Y.-R., Xing Y.-X., Yang L.-T. (2018). Isolation, Transformation and Overexpression of Sugarcane *SoP5CS* Gene for Drought Tolerance Improvement. Sugar Tech.

[107] Mohanan M.V., Pushpanathan A., Padmanabhan S., Sasikumar T., Jayanarayanan A.N., Selvarajan D., Ramalingam S., Ram B., Chinnaswamy A. (2021). Overexpression of Glyoxalase III Gene in Transgenic Sugarcane Confers Enhanced Performance under Salinity Stress. J. Plant Res..

[108] Augustine S.M., Cherian A.V., Syamaladevi D.P., Subramonian N. (2015). *Erianthus arundinaceus* HSP70 (*EaHSP70*) Acts as a Key Regulator in the Formation of Anisotropic Interdigitation in Sugarcane (*Saccharum* spp. Hybrid) in Response to Drought Stress. Plant Cell Physiol..

[109] Belintani N.G., Guerzoni J.T.S., Moreira R.M.P., Vieira L.G.E. (2012). Improving Low-Temperature Tolerance in Sugarcane by Expressing the *Ipt* Gene under a Cold Inducible Promoter. Biol. Plant.

[110] Chen J.-Y., Khan Q., Sun B., Tang L.-H., Yang L.-T., Zhang B.-Q., Xiu X.-Y., Dong D.-F., Li Y.-R. (2021). Overexpression of Sugarcane *SoTUA* Gene Enhances Cold Tolerance in Transgenic Sugarcane. Agron. J..

[111] Zhang S.-Z., Yang B.-P., Feng C.-L., Chen R.-K., Luo J.-P., Cai W.-W., Liu F.-H. (2006). Expression of the *Grifola frondosa* Trehalose Synthase Gene and Improvement of Drought-Tolerance in Sugarcane (*Saccharum officinarum* L.). J. Integr. Plant Biol..

[112] Augustine S.M., Ashwin Narayan J., Syamaladevi D.P., Appunu C., Chakravarthi M., Ravichandran V., Tuteja N., Subramonian N. (2015). Overexpression of *EaDREB2* and Pyramiding of *EaDREB2* with the Pea DNA Helicase Gene (*PDH45*) Enhance Drought and Salinity Tolerance in Sugarcane (*Saccharum* spp. Hybrid). Plant Cell Rep..

[113] Moran J.F., Becana M., Iturbe-Ormaetxe I., Frechilla S., Klucas R.V., Aparicio-Tejo P. (1994). Drought Induces Oxidative Stress in Pea Plants. Planta.

[114] Narayan J.A., Manoj V.M., Nerkar G., Chakravarthi M., Dharshini S., Subramonian N., Premachandran M.N., Valarmathi R., Kumar R.A., Gomathi R. (2023). Transgenic Sugarcane with Higher Levels of *BRK1* Showed Improved Drought Tolerance. Plant Cell Rep..

[115] Mohanan M.V., Thelakat Sasikumar S.P., Jayanarayanan A.N., Selvarajan D., Ramanathan V., Shivalingamurthy S.G., Raju G., Govind H., Chinnaswamy A. (2024). Transgenic Sugarcane Overexpressing *Glyoxalase III* Improved Germination and Biomass Production at Formative Stage under Salinity and Water-Deficit Stress Conditions. 3 Biotech.

[116] Zhao X., Jiang Y., Liu Q., Yang H., Wang Z., Zhang M. (2020). Effects of Drought-Tolerant *Ea-DREB2B* Transgenic Sugarcane on Bacterial Communities in Soil. Front. Microbiol..

[117] Zhao X., Liu Q., Xie S., Jiang Y., Yang H., Wang Z., Zhang M. (2020). Response of Soil Fungal Community to Drought-Resistant *Ea*-*DREB2B* Transgenic Sugarcane. Front. Microbiol..

[118] Mall A.K., Manimekalai R., Misra V., Pandey H., Srivastava S., Sharma A. (2024). CRISPR/Cas-mediated Genome Editing for Sugarcane Improvement. Sugar Tech.

[119] Appunu C., Ram B., Subramonian N., Mohan C. (2017). Sugarcane: An Efficient Platform for Molecular Farming. Sugarcane Biotechnology: Challenges and Prospects.

[120] Fischer R., Stoger E., Schillberg S., Christou P., Twyman R.M. (2004). Plant-Based Production of Biopharmaceuticals. Curr. Opin. Plant Biol..

[121] Jackson M.A., Nutt K.A., Hassall R., Rae A.L. (2010). Comparative Efficiency of Subcellular Targeting Signals for Expression of a Toxic Protein in Sugarcane. Funct. Plant Biol..

[122] Palaniswamy H., Syamaladevi D.P., Mohan C., Philip A., Petchiyappan A., Narayanan S. (2016). Vacuolar Targeting of R-Proteins in Sugarcane Leads to Higher Levels of Purifiable Commercially Equivalent Recombinant Proteins in Cane Juice. Plant Biotechnol. J..

[123] Gabriel C., Fernhout J., Fichtner F., Feil R., Lunn J.E., Kossmann J., Lloyd J.R., van der Vyver C. (2021). Genetic Manipulation of Trehalose-6-phosphate Synthase Results in Changes in the Soluble Sugar Profile in Transgenic Sugarcane Stems. Plant Direct.

[124] Liu H., Lin X., Li X., Luo Z., Lu X., You Q., Yang X., Xu C., Liu X., Liu J. (2023). Haplotype Variations of *Sucrose Phosphate Synthase* Gene among Sugarcane Accessions with Different Sucrose Content. BMC Genom..

[125] Anur R.M., Mufithah N., Sawitri W.D., Sakakibara H., Sugiharto B. (2020). Overexpression of *Sucrose Phosphate Synthase* Enhanced Sucrose Content and Biomass Production in Transgenic Sugarcane. Plants.

[126] Suherman, Wijayanto S.I., Anur R.M., Neliana I.R., Dewanti P., Sugiharto B. (2022). Field Evaluation on Growth and Productivity of the Transgenic Sugarcane Lines Overexpressing Sucrose-Phosphate Synthase. Sugar Technol..

[127] Awan M.F., Ali S., Iqbal M.S., Sharif M.N., Ali Q., Nasir I.A. (2022). Enhancement of Healthful Novel Sugar Contents in Genetically Engineered Sugarcane Juice Integrated with Molecularly Characterized *ThSyGII* (CEMB-SIG2). Sci. Rep..

[128] Petrasovits L.A., Purnell M.P., Nielsen L.K., Brumbley S.M. (2007). Production of Polyhydroxybutyrate in Sugarcane. Plant Biotechnol. J..

[129] Petrasovits L.A., Zhao L., McQualter R.B., Snell K.D., Somleva M.N., Patterson N.A., Nielsen L.K., Brumbley S.M. (2012). Enhanced Polyhydroxybutyrate Production in Transgenic Sugarcane. Plant Biotechnol. J..

[130] Jung J.H., Altpeter F. (2016). TALEN Mediated Targeted Mutagenesis of the Caffeic Acid O-Methyltransferase in Highly Polyploid Sugarcane Improves Cell Wall Composition for Production of Bioethanol. Plant Mol. Biol..

[131] Kannan B., Jung J.H., Moxley G.W., Lee S.-M., Altpeter F. (2018). TALEN-Mediated Targeted Mutagenesis of More than 100 *COMT* Copies/Alleles in Highly Polyploid Sugarcane Improves Saccharification Efficiency without Compromising Biomass Yield. Plant Biotechnol. J..

[132] Jia Y., Maitra S., Singh V. (2023). Chemical-Free Production of Multiple High-Value Bioproducts from Metabolically Engineered Transgenic Sugarcane ‘Oilcane’ Bagasse and Their Recovery Using Nanofiltration. Bioresour. Technol..

[133] Zale J., Jung J.H., Kim J.Y., Pathak B., Karan R., Liu H., Chen X., Wu H., Candreva J., Zhai Z. (2016). Metabolic Engineering of Sugarcane to Accumulate Energy-Dense Triacylglycerols in Vegetative Biomass. Plant Biotechnol. J..

[134] Parajuli S., Kannan B., Karan R., Sanahuja G., Liu H., Garcia-Ruiz E., Kumar D., Singh V., Zhao H., Long S. (2020). Towards Oilcane: Engineering Hyperaccumulation of Triacylglycerol into Sugarcane Stems. GCB Bioenergy.

[135] Bhatti F., Asad S., Khan Q.M., Mobeen A., Iqbal M.J., Asif M. (2019). Risk Assessment of Genetically Modified Sugarcane Expressing *AVP1* Gene. Food Chem. Toxicol..

[136] Perera M.F., Ovejero S.N., Racedo J., Noguera A.S., Cuenya M.I., Castagnaro A.P. (2020). TRAP Markers Allow the Identification of Transgenic Lines That Are Genetically Close to Their Parental Genotype. Sugar Tech.

[137] Schaart J.G., van de Wiel C.C.M., Smulders M.J.M. (2021). Genome Editing of Polyploid Crops: Prospects, Achievements and Bottlenecks. Transgenic Res..

[138] Eid A., Mohan C., Sanchez S., Wang D., Altpeter F. (2021). Multiallelic, Targeted Mutagenesis of Magnesium Chelatase with CRISPR/Cas9 Provides a Rapidly Scorable Phenotype in Highly Polyploid Sugarcane. Front. Genome Ed..

[139] Shabbir R., Javed T., Afzal I., Sabagh A.E., Ali A., Vicente O., Chen P. (2021). Modern Biotechnologies: Innovative and Sustainable Approaches for the Improvement of Sugarcane Tolerance to Environmental Stresses. Agronomy.

[140] Ko J.K., Jung J.H., Altpeter F., Kannan B., Kim H.E., Kim K.H., Alper H.S., Um Y., Lee S.-M. (2018). Largely Enhanced Bioethanol Production through the Combined Use of Lignin-Modified Sugarcane and Xylose Fermenting Yeast Strain. Bioresour. Technol..

[141] Sander J.D., Dahlborg E.J., Goodwin M.J., Cade L., Zhang F., Cifuentes D., Curtin S.J., Blackburn J.S., Thibodeau-Beganny S., Qi Y. (2011). Selection-Free Zinc-Finger-Nuclease Engineering by Context-Dependent Assembly (CoDA). Nat. Methods.

[142] Kumar T., Bao A.-K., Bao Z., Wang F., Gao L., Wang S.-M. (2018). The Progress of Genetic Improvement in Alfalfa (*Medicago sativa* L.). Czech J. Genet. Plant Breed..

[143] Tsanova T., Stefanova L., Topalova L., Atanasov A., Pantchev I. (2021). DNA-Free Gene Editing in Plants: A Brief Overview. Biotechnol. Biotechnol. Equip..

[144] Javaid D., Ganie S.Y., Hajam Y.A., Reshi M.S. (2022). CRISPR/Cas9 System: A Reliable and Facile Genome Editing Tool in Modern Biology. Mol. Biol. Rep..

[145] Wolter F., Puchta H. (2017). Knocking out Consumer Concerns and Regulator’s Rules: Efficient Use of CRISPR/Cas Ribonucleoprotein Complexes for Genome Editing in Cereals. Genome Biol..

[146] Singh R.K., Banerjee N., Khan M.S., Yadav S., Kumar S., Duttamajumder S.K., Lal R.J., Patel J.D., Guo H., Zhang D. (2016). Identification of Putative Candidate Genes for Red Rot Resistance in Sugarcane (*Saccharum* Species Hybrid) Using LD-Based Association Mapping. Mol. Genet. Genom..

[147] Lu S., Zhang H., Guo F., Yang Y., Shen X., Chen B. (2022). *SsUbc2*, a Determinant of Pathogenicity, Functions as a Key Coordinator Controlling Global Transcriptomic Reprogramming during Mating in Sugarcane Smut Fungus. Front. Microbiol..

[148] Zhang H., Yang Y., Guo F., Shen X., Lu S., Chen B. (2023). *SsRSS1* Mediates Salicylic Acid Tolerance and Contributes to Virulence in Sugarcane Smut Fungus. J. Integr. Agric..

[149] Zhang J., Xing J., Mi Q., Yang W., Xiang H., Xu L., Zeng W., Wang J., Deng L., Jiang J. (2023). Highly Efficient Transgene-Free Genome Editing in Tobacco Using an Optimized CRISPR/Cas9 System, pOREU3TR. Plant Sci..

[150] Tyagi S., Kesiraju K., Saakre M., Rathinam M., Raman V., Pattanayak D., Sreevathsa R. (2020). Genome Editing for Resistance to Insect Pests: An Emerging Tool for Crop Improvement. ACS Omega.

[151] Eid A., Mahfouz M.M. (2016). Genome Editing: The Road of CRISPR/Cas9 from Bench to Clinic. Exp. Mol. Med..

[152] Chen Y., Ma J., Zhang X., Yang Y., Zhou D., Yu Q., Que Y., Xu L., Guo J. (2017). A Novel Non-Specific Lipid Transfer Protein Gene from Sugarcane (*NsLTPs*), Obviously Responded to Abiotic Stresses and Signaling Molecules of SA and MeJA. Sugar Tech.

[153] Su Y., Wang Z., Liu F., Li Z., Peng Q., Guo J., Xu L., Que Y. (2016). Isolation and Characterization of *ScGluD2*, a New Sugarcane *Beta*-*1*,*3*-*glucanase* D Family Gene Induced by *Sporisorium scitamineum*, ABA, H_2_O_2_, NaCl, and CdCl_2_ Stresses. Front. Plant Sci..

[154] Wu Q., Pan Y.-B., Su Y., Zou W., Xu F., Sun T., Grisham M.P., Yang S., Xu L., Que Y. (2022). WGCNA Identifies a Comprehensive and Dynamic Gene Co-Expression Network That Associates with Smut Resistance in Sugarcane. Int. J. Mol. Sci..

[155] Luo G., Cao V.D., Kannan B., Liu H., Shanklin J., Altpeter F. (2022). Metabolic Engineering of Energycane to Hyperaccumulate Lipids in Vegetative Biomass. BMC Biotechnol..

[156] Laksana C., Sophiphun O., Chanprame S. (2024). Lignin Reduction in Sugarcane by Performing CRISPR/Cas9 Site-Direct Mutation of *SoLIM* Transcription Factor. Plant Sci..

[157] Kannan B., Liu H., Shanklin J., Altpeter F. (2022). Towards Oilcane: Preliminary Field Evaluation of Metabolically Engineered Sugarcane with Hyper-Accumulation of Triacylglycerol in Vegetative Tissues. Mol. Breed..

[158] Li C., Iqbal M.A. (2024). Leveraging the Sugarcane CRISPR/Cas9 Technique for Genetic Improvement of Non-Cultivated Grasses. Front. Plant Sci..

